# Author Correction: Proteome evolution under non-substitutable resource limitation

**DOI:** 10.1038/s41467-019-12729-x

**Published:** 2019-11-05

**Authors:** Manu Tamminen, Alexander Betz, Aaron Louis Pereira, Marco Thali, Blake Matthews, Marc J.-F. Suter, Anita Narwani

**Affiliations:** 1 Department of Biology, University of Turku, Natura, Yliopistonmäki, 20014 Turku, Finland; 2 Department of Aquatic Ecology, Eawag, Überlandstrasse 133, 8600 Dübendorf, Switzerland; 3 Department of Environmental Toxicology, Eawag, Überlandstrasse 133, 8600 Dübendorf, Switzerland; 4 Department of Aquatic Ecology, Eawag, 79 Seestrasse, 6047 Kastanienbaum, Switzerland

**Keywords:** Evolutionary genetics, Experimental evolution, Metabolism

Correction to: *Nature Communications* 10.1038/s41467-018-07106-z, published online 07 November 2018.

The original version of the Supplementary Information associated with this Article contained several errors. The molar ratio values on the y-axis scale of Supplementary Figure 6 (left panel) were incorrectly labelled to show the data as ranging between 17.2–68.5 rather than between 46.6–185.8 as it should have been. The correct version of Supplementary Figure 6 (left panel) is:



which replaces the previous incorrect version:



Units were missing on the y-axes labels of Supplementary Figure 7 (middle (mg.mg^−1^) and right panels (μg.mg^−1^)). The HTML has been updated to include a corrected version of the Supplementary Information.

