# Supplementary material for: Proteome evolution under non-substitutable resource limitation
Source: Nat Commun. 2018 Nov 7;9:4650. doi: 10.1038/s41467-018-07106-z (PMC6220234; doi:10.1038/s41467-018-07106-z)
Supplement: Supplementary file 1 — Supplementary Information [file 41467_2018_7106_MOESM1_ESM.pdf]

Supplementary Information

Proteome evolution under non-substitutable resource limitation

Tamminen et al.

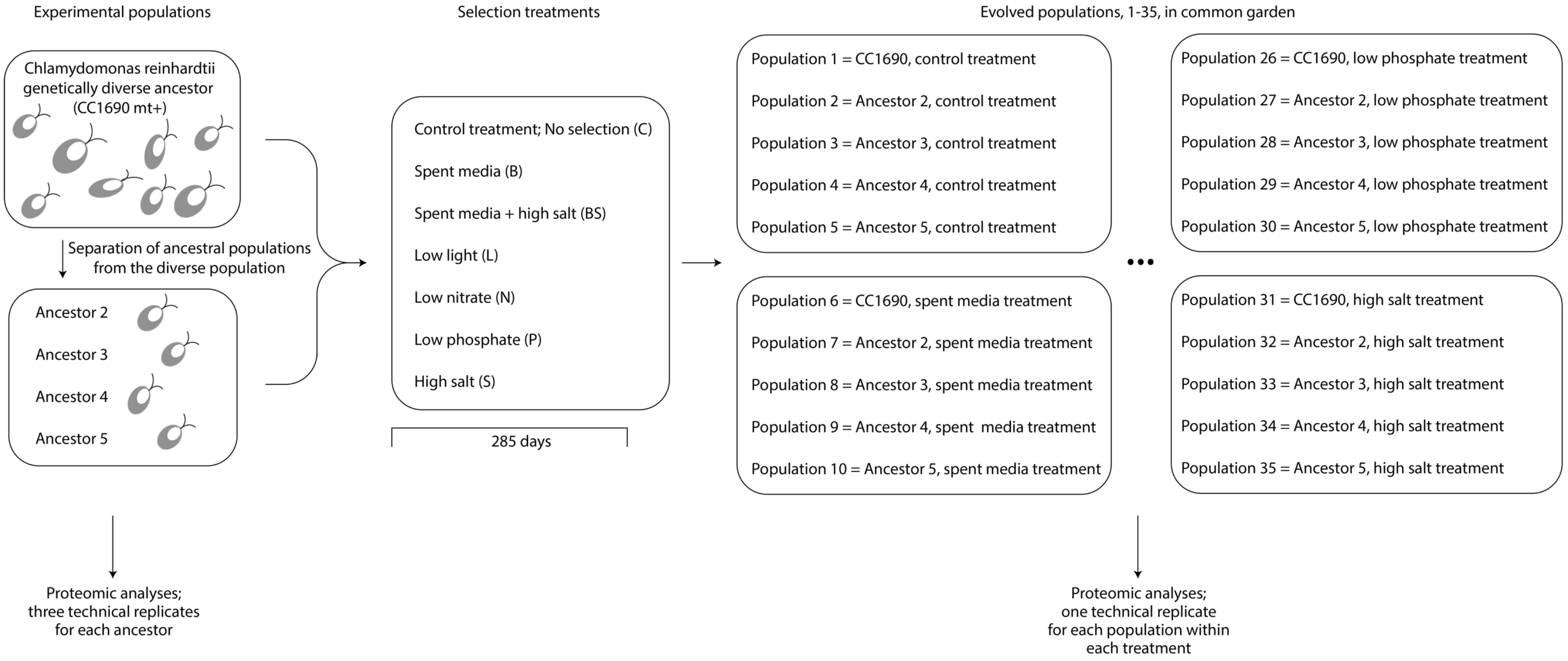

Supplementary Figure 1. The experimental design: four monoclonal populations were isolated from a genetically diverse *Chlamydomonas reinhardtii* ancestor CC1690 mt+. Both the diverse and monoclonal populations were subjected to selection treatments over a period of 285 days, roughly corresponding to 285 generations. After selection, each selected population was grown in common garden for 8 days. Finally, each population from each selection treatment was subjected to proteomic analysis.

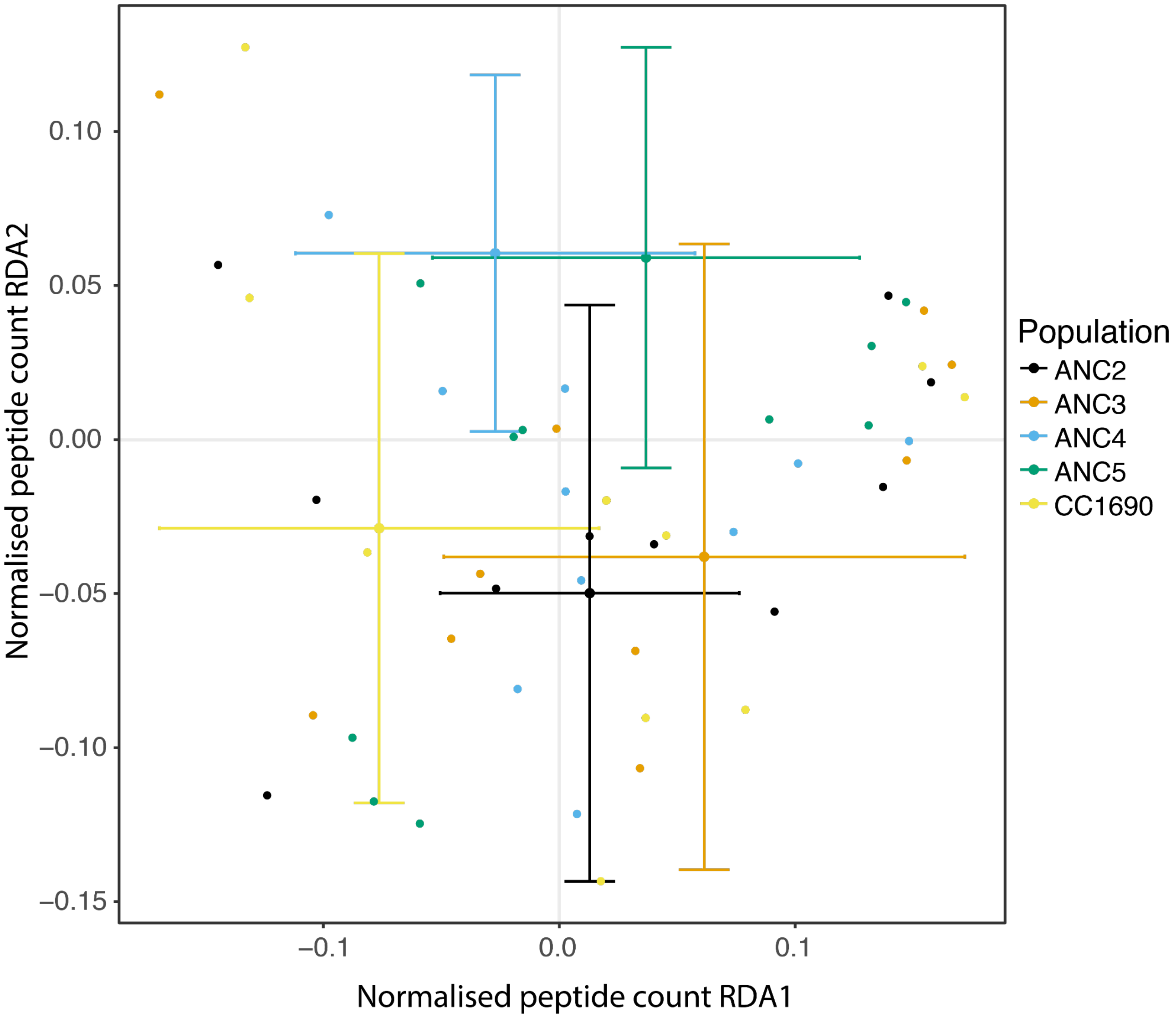

Supplementary Figure 2. The population identity does not account for significant variation in protein expression (redundancy analysis; permutational ANOVA p = 0.762).

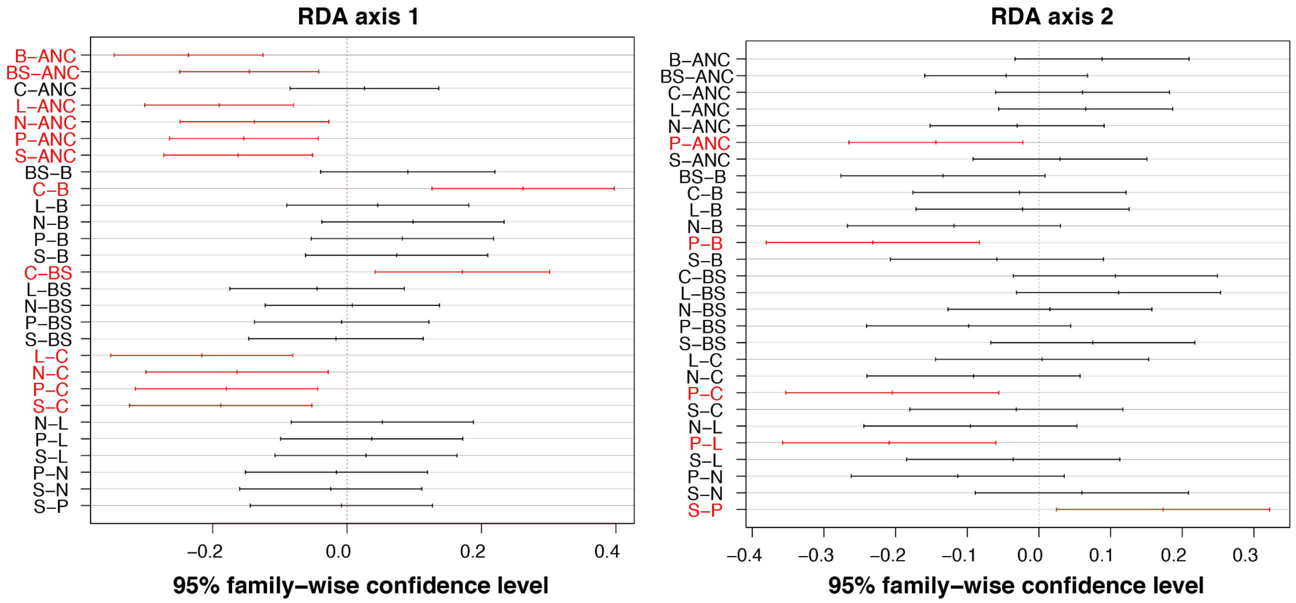

Supplementary Figure 3. Positions of the treatments on the plane of two first RDA axes. Control and ancestral populations are divided into a separate group from all other treatments by RDA axis 1 (ANOVA; TukeyHSD post-doc test; $p < 0.01$). Low-phosphate treatment is divided into a separate group from ancestors, controls, biotic, low-light and high-salt treatments by RDA axis 2 (ANOVA; TukeyHSD post-doc test; $p < 0.01$).

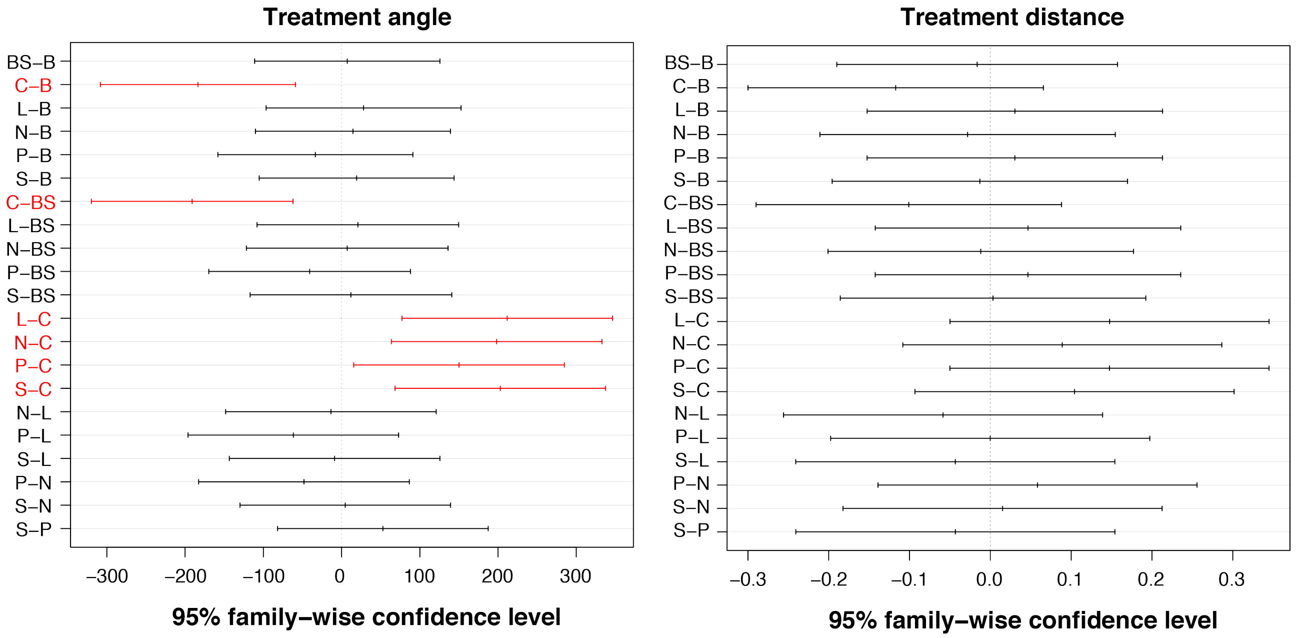

Supplementary Figure 4. Evolutionary distances and directions of the treatments relative to the ancestors. The treatments significantly influence the direction of evolution (ANOVA; $p < 0.001$) grouping the control treatments separately from the other treatments (TukeyHSD post-hoc test; $p < 0.05$). Individual treatments are not significantly different in their distance from the ancestral mean (ANOVA; $p = 0.297$).

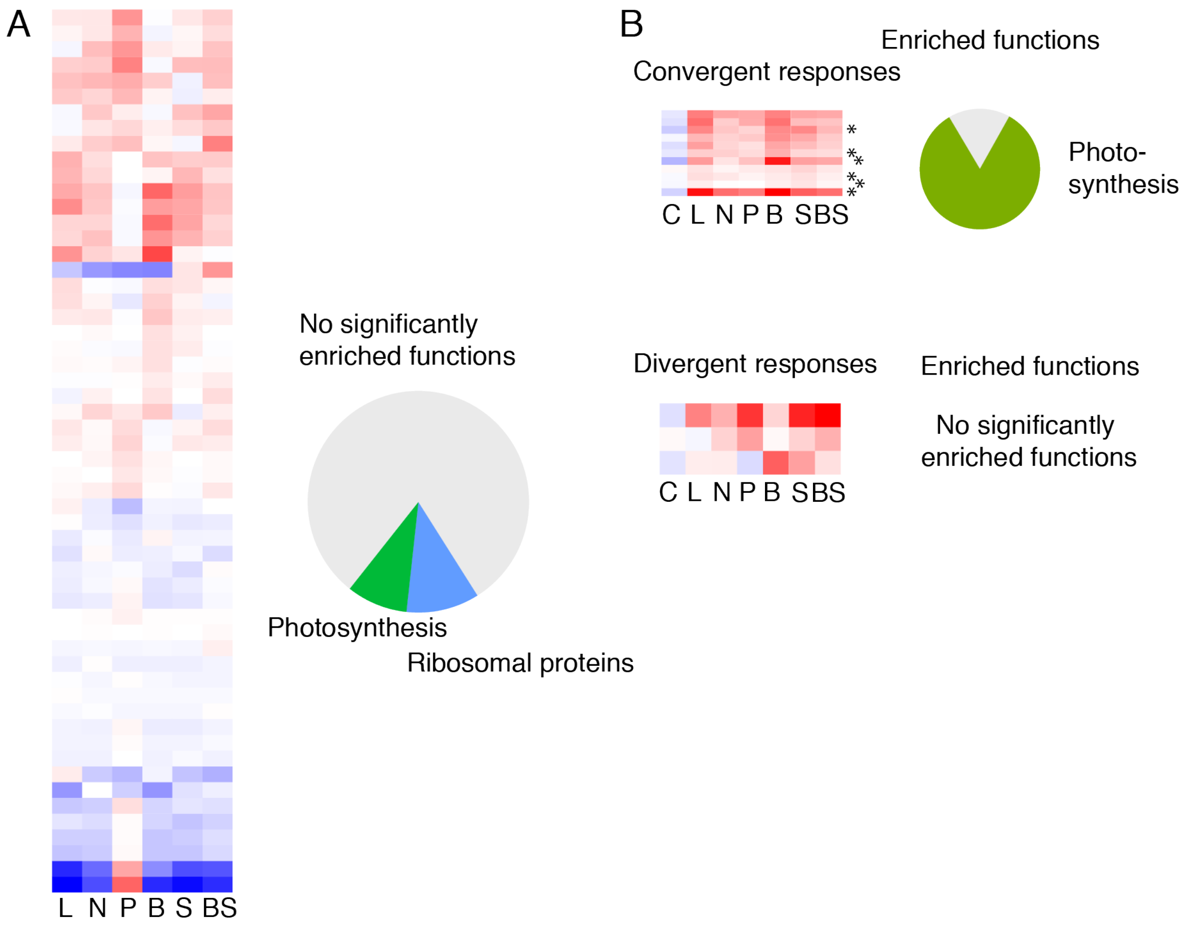

Supplementary Figure 5. A) A total of 56 proteins exhibited an expression pattern where at least one treatment had a different direction from the other treatments. From these proteins, 5 were classified into photosynthesis-related functions and 6 into ribosome-related functions with high significance (DAVID Functional Annotation Clustering; $p < 0.01$). 28 proteins had an annotation in GO Biological Processes database. B) A total of 14 chloroplast-encoded proteins exhibited a significantly different expression level compared to the ancestral populations (t-test $p < 0.001$). Of these, 11 exhibited a convergent (Friedman test $p >= 0.1$) and 4 a divergent response (Friedman test $p < 0.1$). All had an annotation in the GO Biological Processes database.

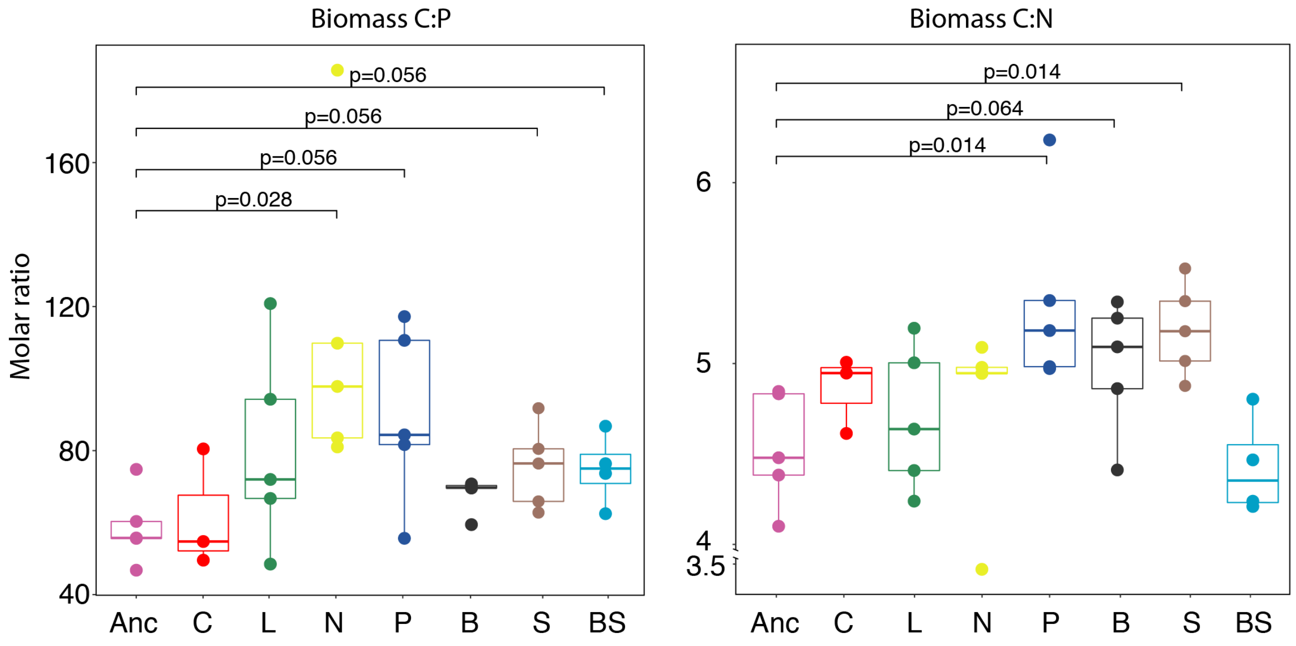

Supplementary Figure 6. Tukey-style boxplots of molar biomass stoichiometry of carbon to phosphorus (C:P) and (C:N) for all ancestors and descendants (according to evolution treatment). The reported p-values are based on Wilcoxon tests testing the alternative hypothesis of treatment median greater than the ancestral median, and adjusted for false discovery rate.

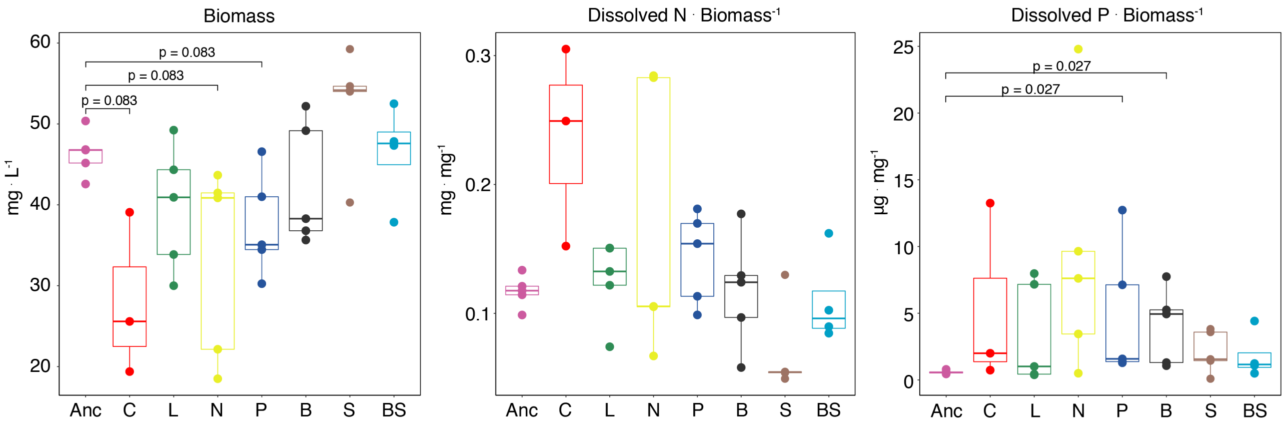

Supplementary Figure 7. Tukey-style boxplots of biomass, mass of dissolved nitrogen remaining in the medium per unit of biomass grown, and mass of dissolved phosphorus remaining in the medium per unit of biomass grown for all ancestors and descendants (according to evolution treatment). The reported p-values are based on Wilcoxon tests testing the alternative two-sided null hypothesis, and adjusted for false discovery rate.

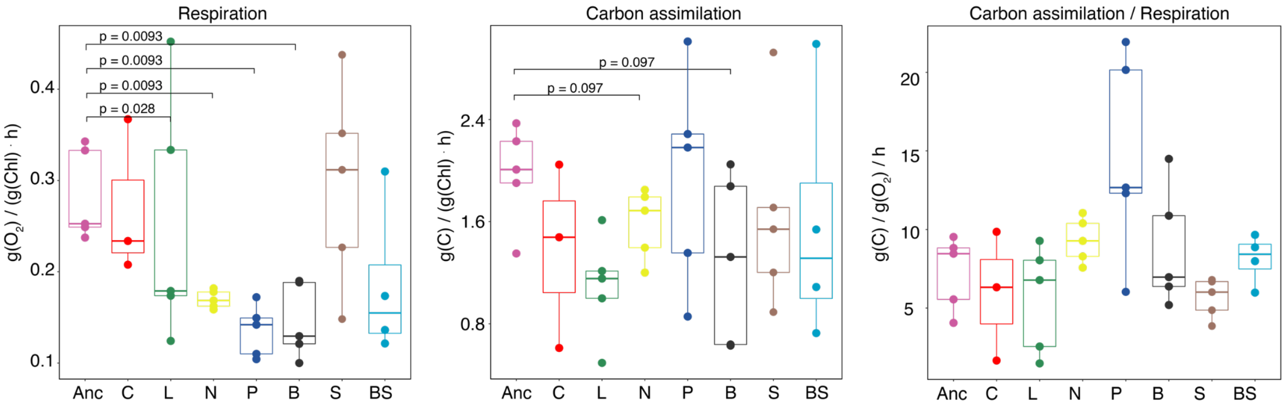

Supplementary Figure 8. Tukey-style boxplots of respiration and photosynthetic carbon assimilation measurements and their ratios for all ancestors and descendants (according to evolution treatment). The reported p-values are based on Wilcoxon tests testing the alternative hypothesis of treatment median less than the ancestral median for respiration and carbon assimilation, and two-sided null-hypothesis for the ratio of carbon assimilation to respiration, and adjusted for false discovery rate.

Species ID	SAG identifier
Cosmarium botrytis	136.8
Kirchneriella subcapitata	12.81
Pediastrum boryanum	87.81
Pediastrum duplex	261-2
Scenedesmus acuminatus	38.81
Staurastrum punctulatum	679-1
Tetraedron minimum	44.81

Supplementary Table 1: The seven species of freshwater algae grown on COMBO to produce the biotically-depleted medium ('Biotic'). SAG = The Culture Collection of Algae at Goettingen University.
