# Supplementary material for: Proteome evolution under non-substitutable resource limitation
Source: Nat Commun. 2018 Nov 7;9:4650. doi: 10.1038/s41467-018-07106-z (PMC6220234; doi:10.1038/s41467-018-07106-z)
Supplement: Supplementary file 3 — Description of Additional Supplementary Files [file 41467_2018_7106_MOESM3_ESM.pdf]

Description of Additional Supplementary Files

File Name: Supplementary Data 1

Description: Core metabolic pathways of *C. reinhardtii*. The blank circles represent metabolites and the square boxes enzymes,with blue indicating down-regulation and red up-regulation, and the embedded numbers corresponding to Enzyme Catalogue (EC) numbers. A-B) Photosynthetic machinery. C) Protein synthetic machinery. D-E) Fatty acid metabolism. F) Pyrimidine metabolism. G) Purine metabolism. H-V) Amino acid metabolism.
