# Supplementary material for: Proteome evolution under non-substitutable resource limitation
Source: Nat Commun. 2018 Nov 7;9:4650. doi: 10.1038/s41467-018-07106-z (PMC6220234; doi:10.1038/s41467-018-07106-z)
Supplement: Supplementary file 4 — Supplementary Data 1 [file 41467_2018_7106_MOESM4_ESM.pdf]

A)

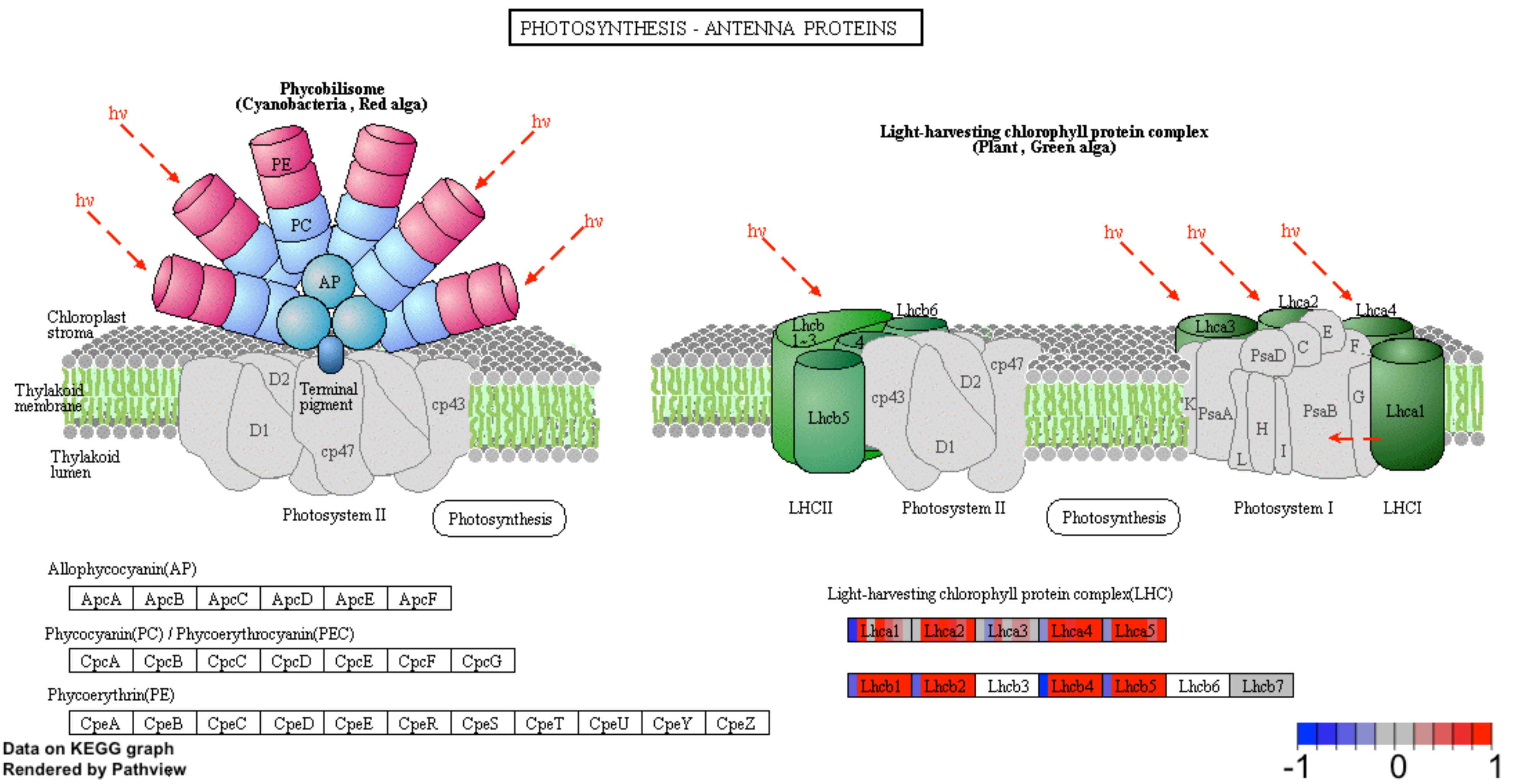
PHOTOSYNTHESIS - ANTENNA PROTEINS
Phycobilisome
(Cyanobacteria , Red alga)
hv
PE
PC
AP
Chloroplast stroma
Thylakoid membrane
Thylakoid lumen
D2
D1
Terminal pigment
cp43
cp47
Photosystem II
Photosynthesis
Light-harvesting chlorophyll protein complex
(Plant , Green alga)
Lhcb 1~3
Lhcb6
Lhcb5
Lhca3
Lhca2
Lhca4
Lhca1
PsaD
PsaA
PsaB
C
E
F
G
H
I
K
L
LHCII
Photosystem II
Photosynthesis
Photosystem I
LHCI
Allophycocyanin(AP)
ApcA
ApcB
ApcC
ApcD
ApcE
ApcF
Phycocyanin(PC) / Phycoerythrocyanin(PEC)
CpcA
CpcB
CpcC
CpcD
CpcE
CpcF
CpcG
Phycoerythrin(PE)
CpeA
CpeB
CpeC
CpeD
CpeE
CpeR
CpeS
CpeT
CpeU
CpeY
CpeZ
Light-harvesting chlorophyll protein complex(LHC)
Lhca1
Lhca2
Lhca3
Lhca4
Lhca5
Lhcb1
Lhcb2
Lhcb3
Lhcb4
Lhcb5
Lhcb6
Lhcb7
-1
0
1
Data on KEGG graph
Rendered by Pathview

B)

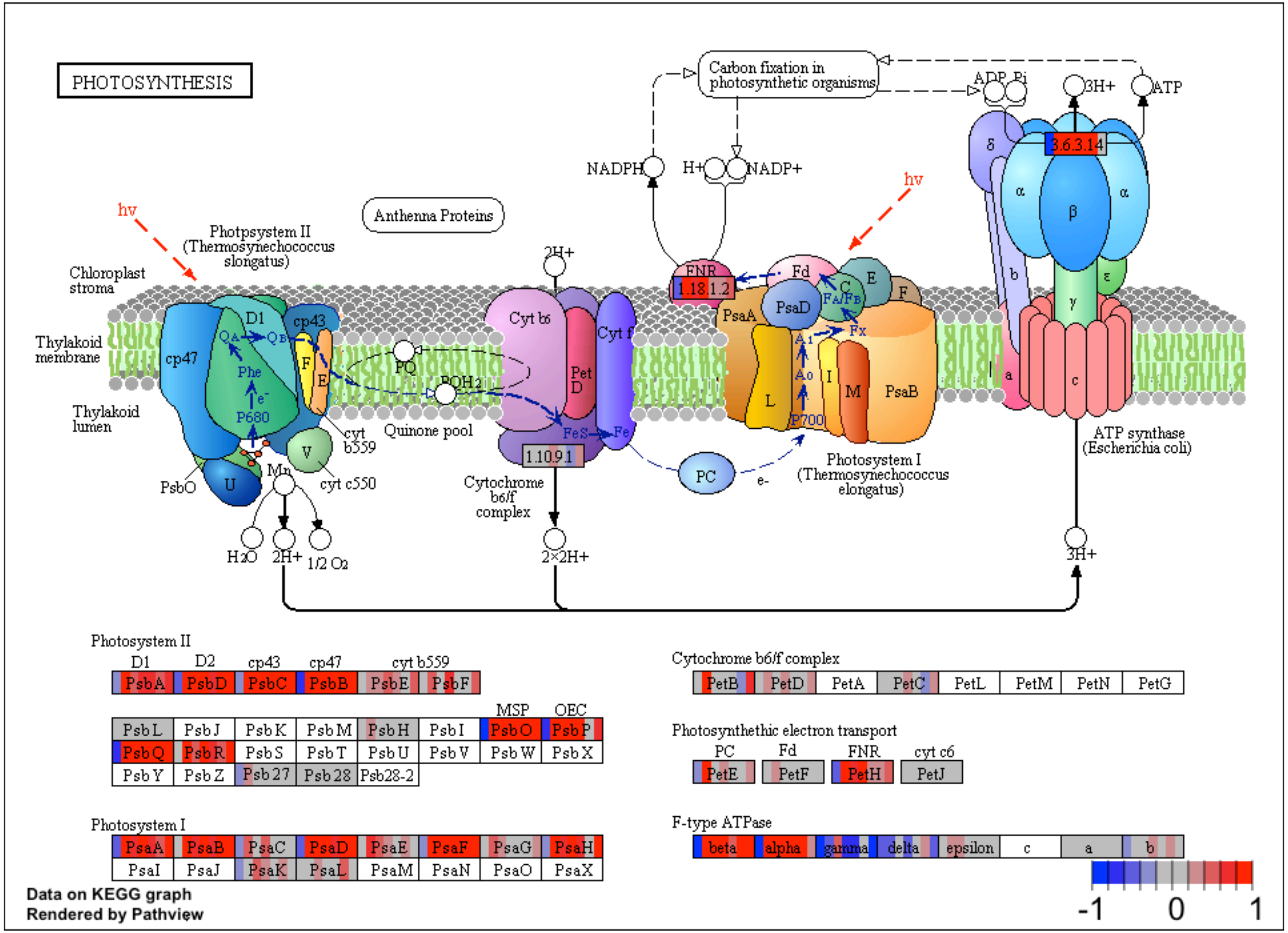

PHOTOSYNTHESIS
Carbon fixation in photosynthetic organisms
ADP Pi
3H+
ATP
NADPH
H+
NADP+
3.6.3.14
hv
Anthenna Proteins
Photpsystem II (Thermosynechococcus elongatus)
Chloroplast stroma
Thylakoid membrane
Thylakoid lumen
D1
cp43
cp47
PQ
PQH2
Quinone pool
cyt b559
cyt c550
PsbO
U
V
Mn
H2O
2H+
1/2 O2
Cyt b6
Pet D
Cyt f
1.10.9.1
Cytochrome b6/f complex
2×2H+
FNR
1.18.1.2
Fd
PsaD
PsaA
PsaB
L
I
M
E
F
PC
e-
P700
Photosystem I (Thermosynechococcus elongatus)
ATP synthase (Escherichia coli)
Photosystem II
D1 D2 cp43 cp47 cyt b559
PsbA PsbD PsbC PsbB PsbE PsbF
MSP OEC
PsbL PsbJ PsbK PsbM PsbH PsbI PsbO PsbP
PsbQ PsbR PsbS PsbT PsbU PsbV PsbW PsbX
PsbY PsbZ Psb27 Psb28 Psb28-2
Photosystem I
PsaA PsaB PsaC PsaD PsaE PsaF PsaG PsaH
PsaI PsaJ PsaK PsaL PsaM PsaN PsaO PsaX
Cytochrome b6/f complex
PetB PetD PetA PetC PetL PetM PetN PetG
Photosynthethic electron transport
PC Fd FNR cyt c6
PetE PetF PetH PetJ
F-type ATPase
beta alpha gamma delta epsilon c a b
-1 0 1
Data on KEGG graph
Rendered by Pathview

C)

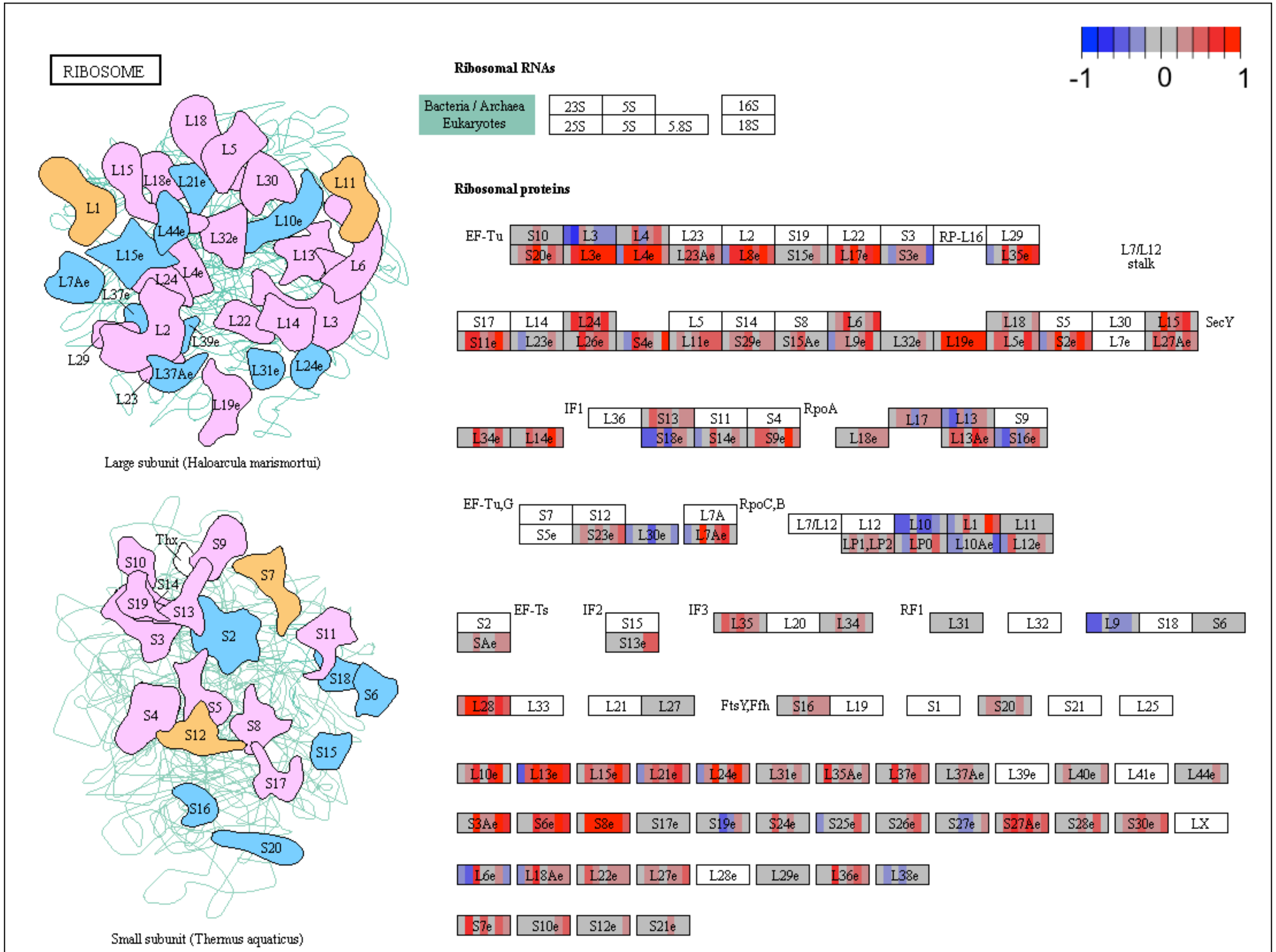

Data on KEGG graph
Rendered by Pathview

D)

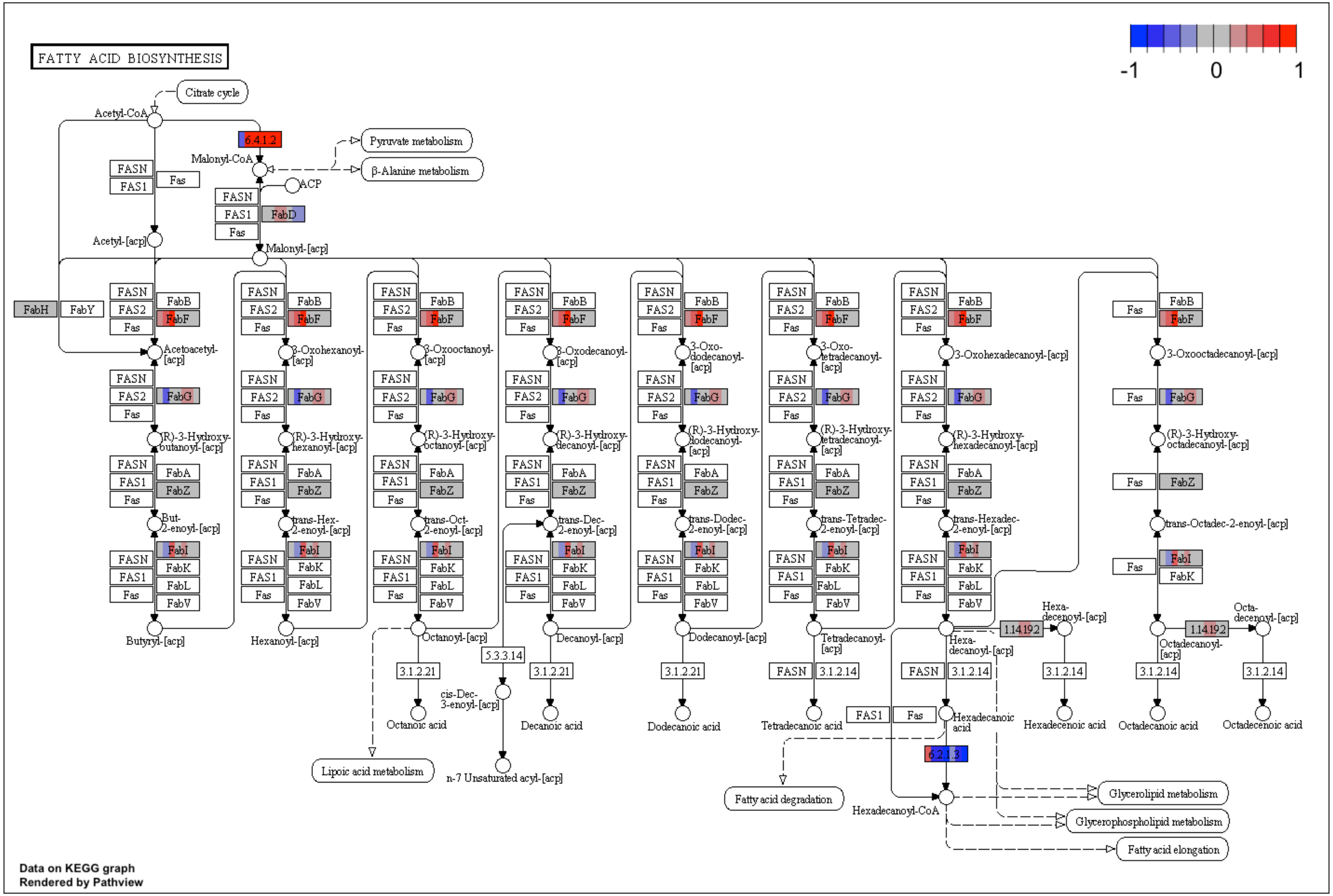
FATTY ACID BIOSYNTHESIS
-1
0
1
Citrate cycle
Acetyl-CoA
6.4.1.2
Pyruvate metabolism
Malonyl-CoA
β-Alanine metabolism
ACP
FASN
FAS1
FAS2
Fas
FabD
Acetyl-[acp]
Malonyl-[acp]
FabH
FabY
FabB
FabF
FabG
FabA
FabZ
FabI
FabK
FabL
FabV
Acetoacetyl-[acp]
(R)-3-Hydroxy-butanoyl-[acp]
But-2-enoyl-[acp]
Butyryl-[acp]
3-Oxohexanoyl-[acp]
(R)-3-Hydroxy-hexanoyl-[acp]
trans-Hex-2-enoyl-[acp]
Hexanoyl-[acp]
3-Oxooctanoyl-[acp]
(R)-3-Hydroxy-octanoyl-[acp]
trans-Oct-2-enoyl-[acp]
Octanoyl-[acp]
3-Oxodecanoyl-[acp]
(R)-3-Hydroxy-decanoyl-[acp]
trans-Dec-2-enoyl-[acp]
Decanoyl-[acp]
3-Oxo-dodecanoyl-[acp]
(R)-3-Hydroxy-dodecanoyl-[acp]
trans-Dodec-2-enoyl-[acp]
Dodecanoyl-[acp]
3-Oxo-tetradecanoyl-[acp]
(R)-3-Hydroxy-tetradecanoyl-[acp]
trans-Tetradec-2-enoyl-[acp]
Tetradecanoyl-[acp]
3-Oxohexadecanoyl-[acp]
(R)-3-Hydroxy-hexadecanoyl-[acp]
trans-Hexadec-2-enoyl-[acp]
Hexa-decanoyl-[acp]
3-Oxooctadecanoyl-[acp]
(R)-3-Hydroxy-octadecanoyl-[acp]
trans-Octadec-2-enoyl-[acp]
Octadecanoyl-[acp]
1.14.19.2
Hexa-decenoyl-[acp]
Octa-decenoyl-[acp]
5.3.3.14
cis-Dec-3-enoyl-[acp]
3.1.2.21
3.1.2.14
Octanoic acid
Decanoic acid
Dodecanoic acid
Tetradecanoic acid
Hexadecanoic acid
Hexadecenoic acid
Octadecanoic acid
Octadecenoic acid
Lipoic acid metabolism
n-7 Unsaturated acyl-[acp]
Fatty acid degradation
6.2.1.3
Hexadecanoyl-CoA
Glycerolipid metabolism
Glycerophospholipid metabolism
Fatty acid elongation
Data on KEGG graph
Rendered by Pathview

E)

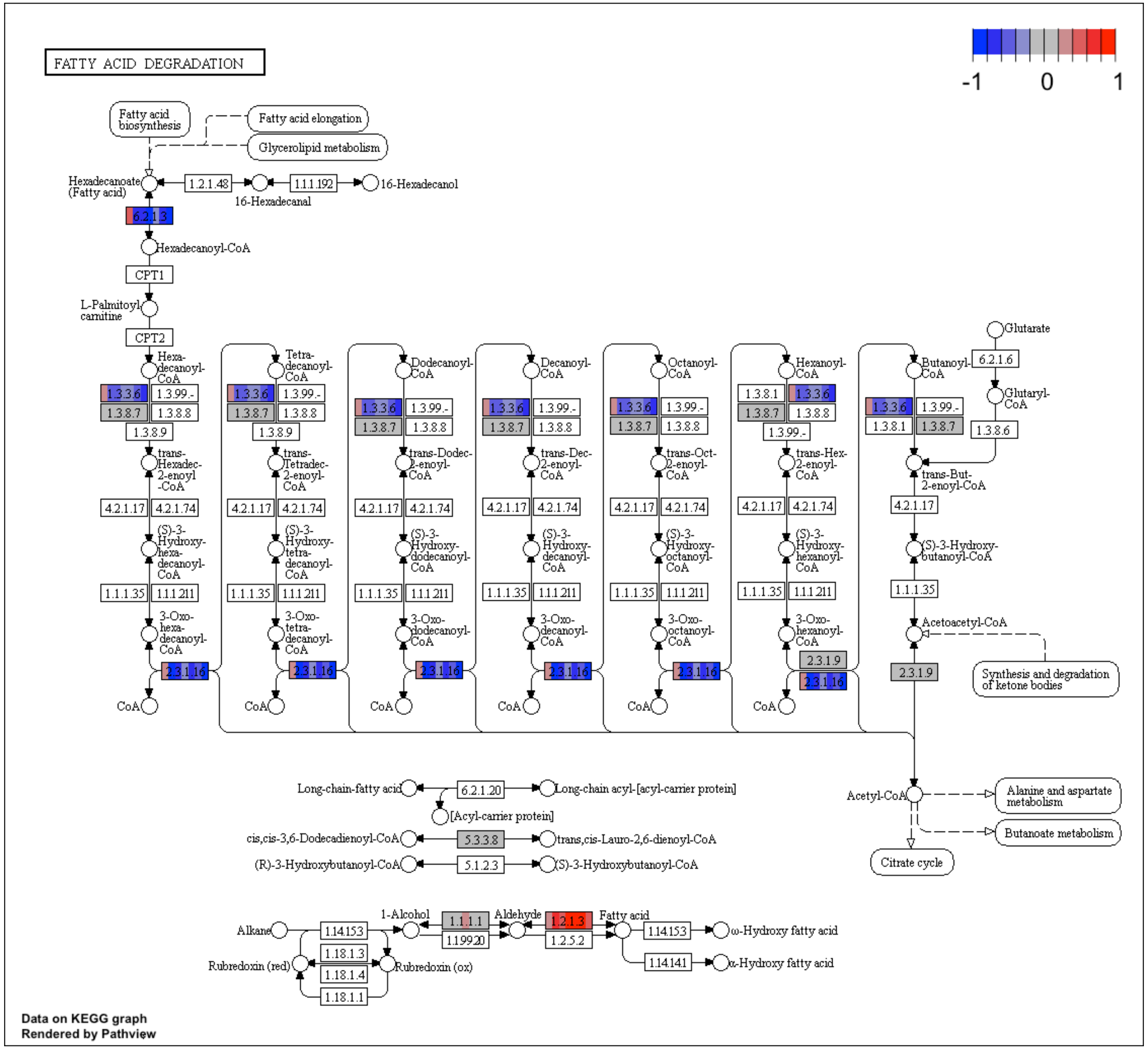

FATTY ACID DEGRADATION
-1
0
1
Fatty acid biosynthesis
Fatty acid elongation
Glycerolipid metabolism
Hexadecanoate (Fatty acid)
1.2.1.48
1.1.1.192
16-Hexadecanol
16-Hexadecanal
6.2.1.3
Hexadecanoyl-CoA
CPT1
L-Palmitoyl-carnitine
CPT2
Hexa-decanoyl-CoA
Tetra-decanoyl-CoA
Dodecanoyl-CoA
Decanoyl-CoA
Octanoyl-CoA
Hexanoyl-CoA
Butanoyl-CoA
Glutarate
6.2.1.6
Glutaryl-CoA
1.3.8.6
1.3.3.6
1.3.99.-
1.3.8.7
1.3.8.8
1.3.8.9
1.3.8.1
trans-Hexadec-2-enoyl-CoA
trans-Tetradec-2-enoyl-CoA
trans-Dodec-2-enoyl-CoA
trans-Dec-2-enoyl-CoA
trans-Oct-2-enoyl-CoA
trans-Hex-2-enoyl-CoA
trans-But-2-enoyl-CoA
4.2.1.17
4.2.1.74
(S)-3-Hydroxy-hexa-decanoyl-CoA
(S)-3-Hydroxy-tetra-decanoyl-CoA
(S)-3-Hydroxy-dodecanoyl-CoA
(S)-3-Hydroxy-decanoyl-CoA
(S)-3-Hydroxy-octanoyl-CoA
(S)-3-Hydroxy-hexanoyl-CoA
(S)-3-Hydroxy-butanoyl-CoA
1.1.1.35
1.1.1.211
3-Oxo-hexa-decanoyl-CoA
3-Oxo-tetra-decanoyl-CoA
3-Oxo-dodecanoyl-CoA
3-Oxo-decanoyl-CoA
3-Oxo-octanoyl-CoA
3-Oxo-hexanoyl-CoA
Acetoacetyl-CoA
2.3.1.16
2.3.1.9
CoA
Synthesis and degradation of ketone bodies
Acetyl-CoA
Alanine and aspartate metabolism
Butanoate metabolism
Citrate cycle
Long-chain-fatty acid
6.2.1.20
Long-chain acyl-[acyl-carrier protein]
[Acyl-carrier protein]
cis,cis-3,6-Dodecadienoyl-CoA
5.3.3.8
trans,cis-Lauro-2,6-dienoyl-CoA
(R)-3-Hydroxybutanoyl-CoA
5.1.2.3
(S)-3-Hydroxybutanoyl-CoA
Alkane
1.14.15.3
1.18.1.3
1.18.1.4
1.18.1.1
Rubredoxin (red)
Rubredoxin (ox)
1-Alcohol
1.1.1.1
1.1.99.20
Aldehyde
1.2.1.3
1.2.5.2
Fatty acid
1.14.15.3
ω-Hydroxy fatty acid
1.14.14.1
α-Hydroxy fatty acid
Data on KEGG graph
Rendered by Pathview

F)

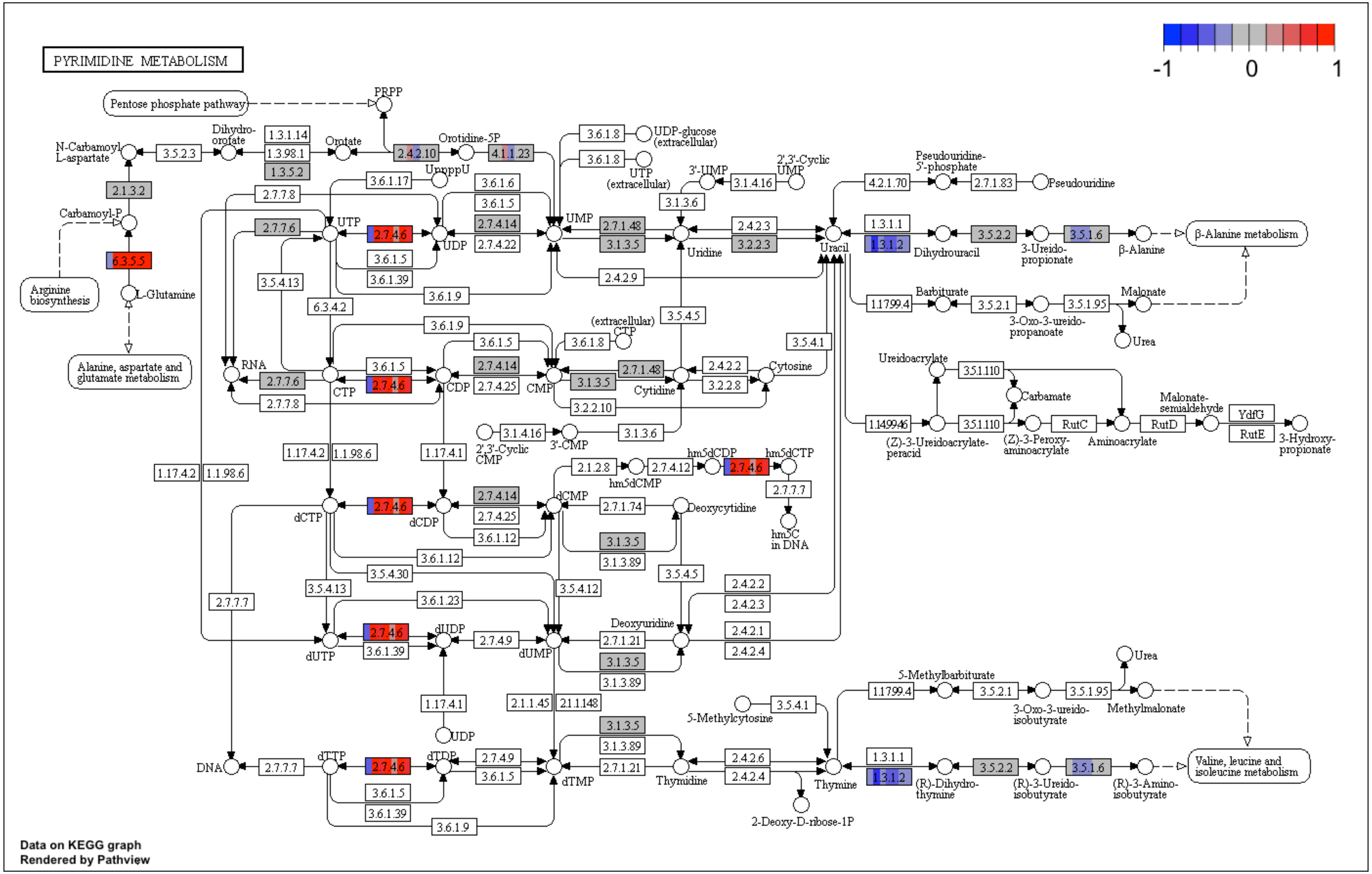
PYRIMIDINE METABOLISM
-1
0
1
Pentose phosphate pathway
PRPP
N-Carbamoyl-L-aspartate
3.5.2.3
Dihydro-orotate
1.3.1.14
1.3.98.1
1.3.5.2
Orotate
2.4.2.10
Orotidine-5P
4.1.1.23
2.1.3.2
Carbamoyl-P
6.3.5.5
L-Glutamine
Arginine biosynthesis
Alanine, aspartate and glutamate metabolism
3.6.1.17
UpppU
2.7.7.8
2.7.7.6
UTP
2.7.4.6
UDP
3.6.1.6
3.6.1.5
2.7.4.14
2.7.4.22
UMP
3.6.1.39
3.6.1.9
3.5.4.13
6.3.4.2
RNA
CTP
CDP
CMP
3.6.1.8
UDP-glucose (extracellular)
UTP (extracellular)
2.7.1.48
3.1.3.5
3.1.3.6
3'-UMP
3.1.4.16
2',3'-Cyclic UMP
Uridine
2.4.2.3
3.2.2.3
2.4.2.9
Uracil
Pseudouridine-5'-phosphate
4.2.1.70
2.7.1.83
Pseudouridine
1.3.1.1
1.3.1.2
Dihydrouracil
3.5.2.2
3-Ureido-propionate
3.5.1.6
β-Alanine
β-Alanine metabolism
1.17.99.4
Barbiturate
3.5.2.1
3-Oxo-3-ureido-propanoate
3.5.1.95
Malonate
Urea
(extracellular) CTP
3.5.4.5
3.5.4.1
2.4.2.2
3.2.2.8
Cytosine
Cytidine
3.2.2.10
2.7.4.25
Ureidoacrylate
3.5.1.110
Carbamate
1.14.99.46
(Z)-3-Ureidoacrylate-peracid
(Z)-3-Peroxy-aminoacrylate
RutC
Aminoacrylate
RutD
Malonate-semialdehyde
YdfG
RutE
3-Hydroxy-propionate
2',3'-Cyclic CMP
3'-CMP
1.17.4.2
1.1.98.6
1.17.4.1
2.1.2.8
hm5dCMP
2.7.4.12
hm5dCDP
hm5dCTP
2.7.7.7
hm5C in DNA
dCTP
dCDP
dCMP
2.7.1.74
Deoxycytidine
3.6.1.12
3.1.3.89
3.5.4.30
3.6.1.23
3.5.4.12
dUTP
dUDP
2.7.4.9
dUMP
Deoxyuridine
2.7.1.21
2.4.2.1
2.4.2.4
2.1.1.45
2.1.1.148
UDP
5-Methylbarbiturate
3-Oxo-3-ureido-isobutyrate
Methylmalonate
5-Methylcytosine
Valine, leucine and isoleucine metabolism
DNA
dTTP
dTDP
dTMP
Thymidine
2.4.2.6
Thymine
2-Deoxy-D-ribose-1P
(R)-Dihydro-thymine
(R)-3-Ureido-isobutyrate
(R)-3-Amino-isobutyrate
Data on KEGG graph
Rendered by Pathview

G)

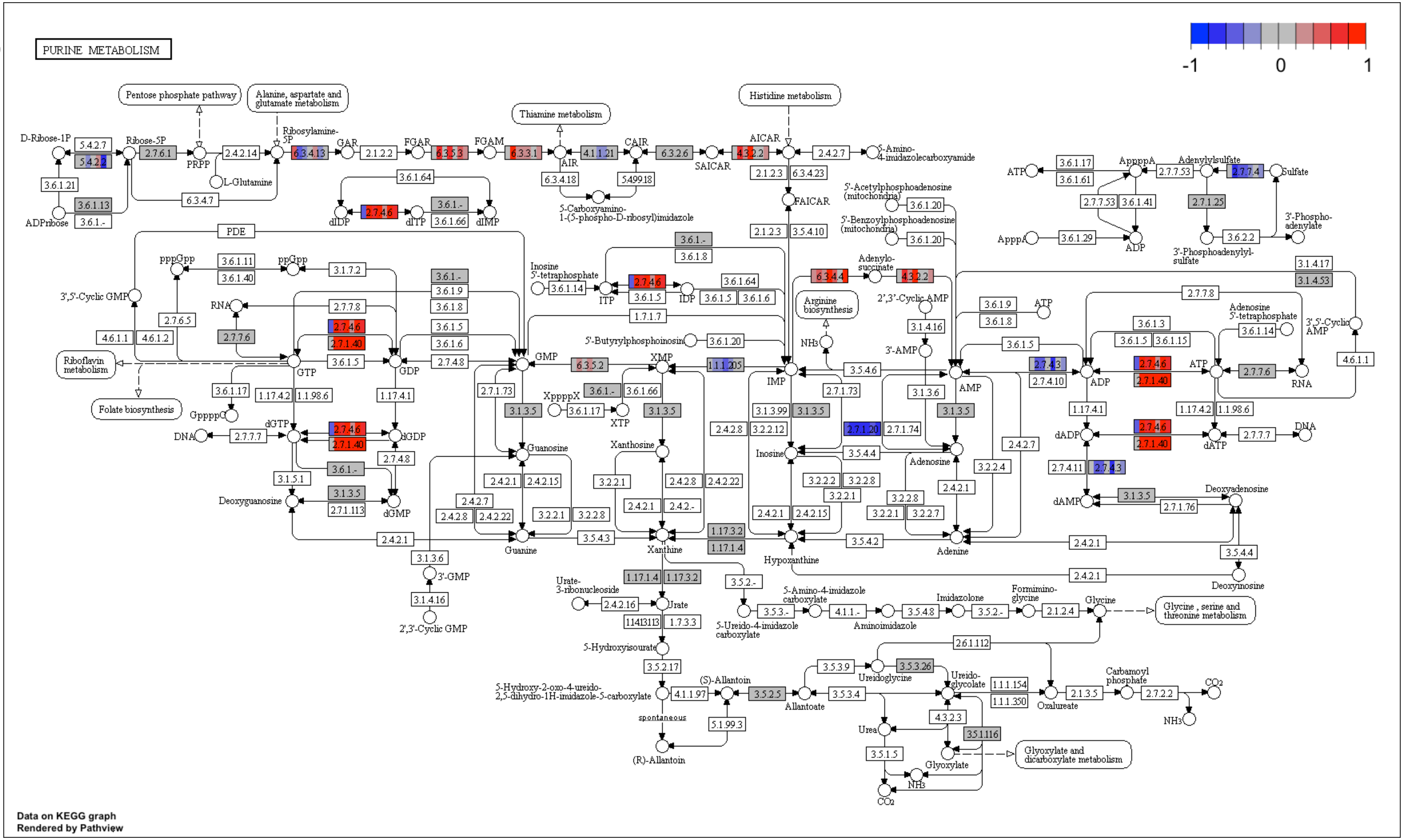
PURINE METABOLISM
-1
0
1
Pentose phosphate pathway
Alanine, aspartate and glutamate metabolism
Thiamine metabolism
Histidine metabolism
D-Ribose-1P
Ribose-5P
PRPP
Ribosylamine-5P
GAR
FGAR
FGAM
AIR
CAIR
SAICAR
AICAR
5-Amino-4-imidazolecarboxyamide
L-Glutamine
ADPribose
5-Carboxyamino-1-(5-phospho-D-ribosyl)imidazole
FAICAR
dIDP
dITP
dIMP
PDE
pppGpp
ppGpp
3',5'-Cyclic GMP
RNA
GTP
GDP
GMP
Riboflavin metabolism
Folate biosynthesis
GppppG
DNA
dGTP
dGDP
dGMP
Deoxyguanosine
Inosine 5'-tetraphosphate
ITP
IDP
IMP
5'-Butyrylphosphoinosine
XMP
XppppX
XTP
Xanthosine
Guanosine
Guanine
Xanthine
Hypoxanthine
Inosine
Adenosine
Adenine
AMP
3'-AMP
2',3'-Cyclic AMP
Adenylo-succinate
Arginine biosynthesis
NH3
5'-Acetylphosphoadenosine (mitochondria)
5'-Benzoylphosphoadenosine (mitochondria)
ATP
ApppA
AppppA
ADP
Adenylylsulfate
Sulfate
3'-Phospho-adenylate
3'-Phosphoadenylyl-sulfate
Adenosine 5'-tetraphosphate
3',5'-Cyclic AMP
RNA
dADP
dATP
dAMP
DNA
Deoxyadenosine
Deoxyinosine
3'-GMP
2',3'-Cyclic GMP
Urate-3-ribonucleoside
Urate
5-Hydroxyisourate
5-Hydroxy-2-oxo-4-ureido-2,5-dihydro-1H-imidazole-5-carboxylate
(S)-Allantoin
(R)-Allantoin
spontaneous
5-Amino-4-imidazole carboxylate
5-Ureido-4-imidazole carboxylate
Aminoimidazolone
Imidazolone
Formimino-glycine
Glycine
Glycine, serine and threonine metabolism
Allantoate
Ureidoglycine
Ureido-glycolate
Urea
Glyoxylate
Glyoxylate and dicarboxylate metabolism
Oxalureate
Carbamoyl phosphate
CO2
NH3
Data on KEGG graph
Rendered by Pathview

H)

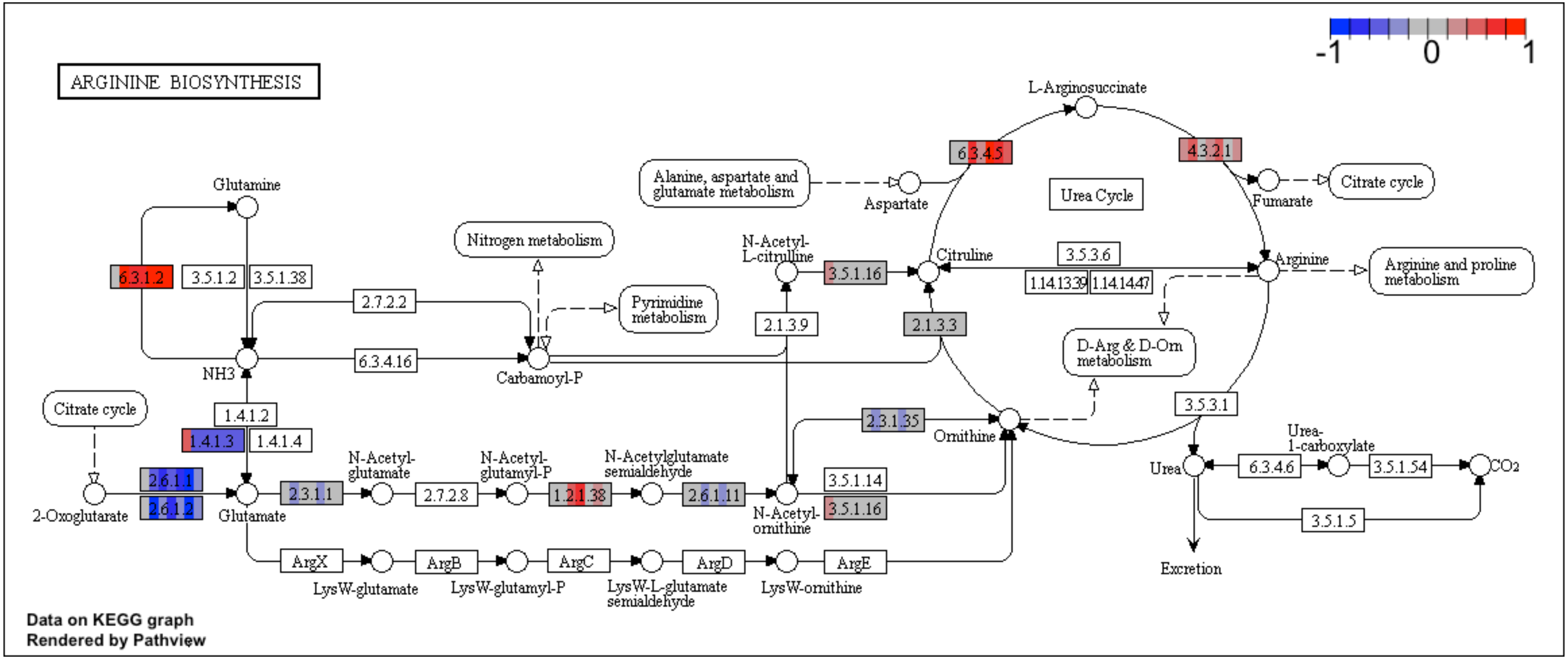

ARGININE BIOSYNTHESIS
-1
0
1
L-Arginosuccinate
6.3.4.5
4.3.2.1
Alanine, aspartate and glutamate metabolism
Aspartate
Citrate cycle
Fumarate
Urea Cycle
Glutamine
Nitrogen metabolism
N-Acetyl-L-citrulline
Citrulline
3.5.3.6
Arginine
Arginine and proline metabolism
6.3.1.2
3.5.1.2
3.5.1.38
3.5.1.16
1.14.13.39
1.14.14.47
2.7.2.2
Pyrimidine metabolism
2.1.3.9
2.1.3.3
D-Arg & D-Orn metabolism
6.3.4.16
NH3
Carbamoyl-P
Citrate cycle
1.4.1.2
3.5.3.1
2.3.1.35
1.4.1.3
1.4.1.4
Ornithine
Urea-1-carboxylate
N-Acetyl-glutamate
N-Acetyl-glutamyl-P
N-Acetylglutamate semialdehyde
2.6.1.1
3.5.1.14
Urea
6.3.4.6
3.5.1.54
CO2
2-Oxoglutarate
2.6.1.2
Glutamate
2.3.1.1
2.7.2.8
1.2.1.38
2.6.1.11
N-Acetyl-ornithine
3.5.1.16
3.5.1.5
ArgX
ArgB
ArgC
ArgD
ArgE
Excretion
LysW-glutamate
LysW-glutamyl-P
LysW-L-glutamate semialdehyde
LysW-ornithine
Data on KEGG graph
Rendered by Pathview

I)

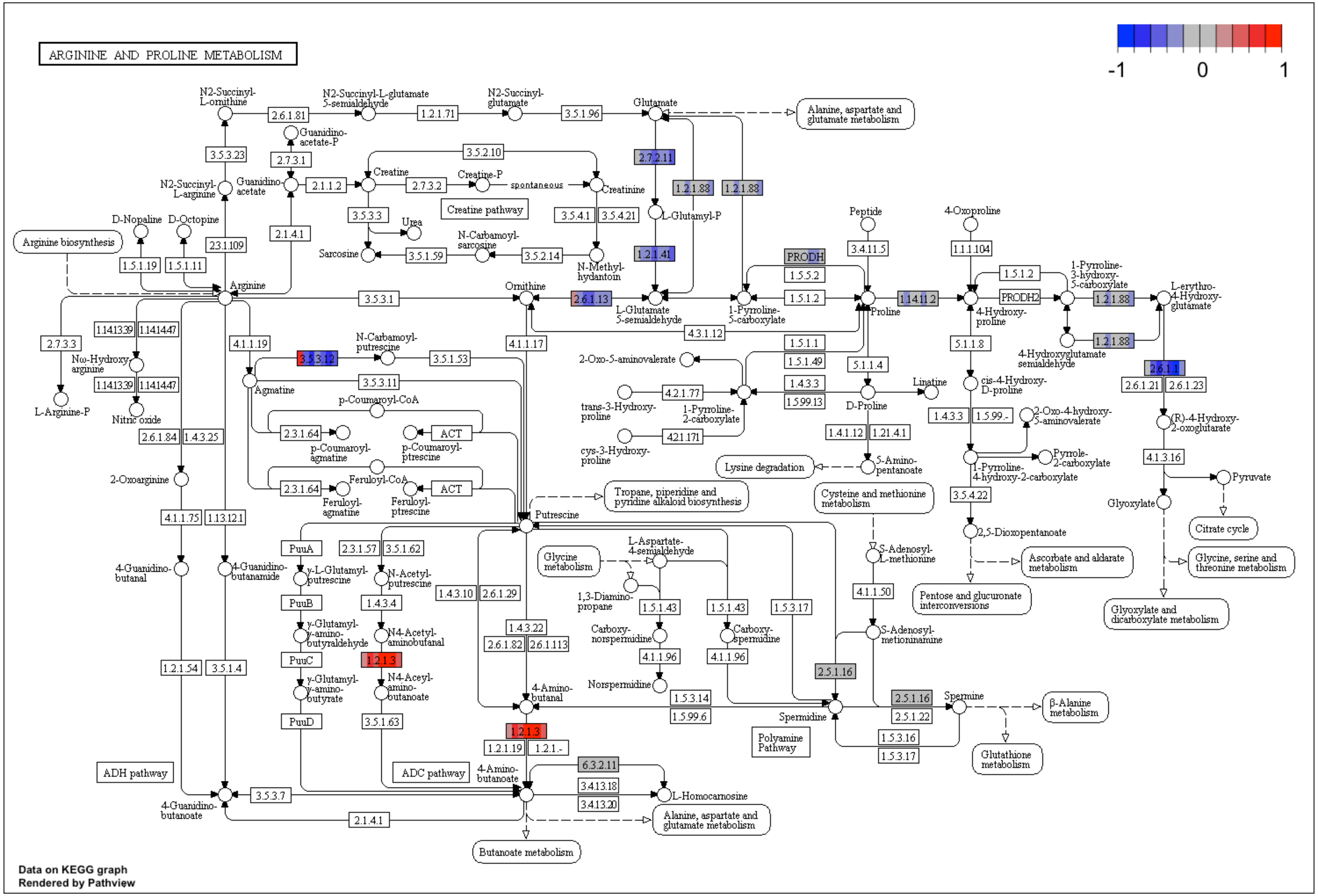
ARGININE AND PROLINE METABOLISM
-1
0
1
N2-Succinyl-L-ornithine
2.6.1.81
N2-Succinyl-L-glutamate 5-semialdehyde
1.2.1.71
N2-Succinyl-glutamate
3.5.1.96
Glutamate
Alanine, aspartate and glutamate metabolism
3.5.3.23
Guanidino-acetate-P
2.7.3.1
3.5.2.10
2.7.2.11
1.2.1.88
1.2.1.88
N2-Succinyl-L-arginine
Guanidino-acetate
2.1.1.2
Creatine
2.7.3.2
Creatine-P
spontaneous
Creatinine
Creatine pathway
D-Nopaline
D-Octopine
3.5.3.3
Urea
3.5.4.1
3.5.4.21
L-Glutamyl-P
Peptide
4-Oxoproline
2.3.1.109
2.1.4.1
N-Carbamoyl-sarcosine
Arginine biosynthesis
1.5.1.19
1.5.1.11
Sarcosine
3.5.1.59
3.5.2.14
N-Methyl-hydantoin
1.2.1.41
PRODH
3.4.11.5
1.1.1.104
1.5.5.2
1.5.1.2
1-Pyrroline-3-hydroxy-5-carboxylate
Arginine
Ornithine
L-erythro-4-Hydroxy-glutamate
3.5.3.1
2.6.1.13
L-Glutamate 5-semialdehyde
1.5.1.2
1-Pyrroline-5-carboxylate
Proline
1.14.11.2
PRODH2
1.2.1.88
4-Hydroxy-proline
2.7.3.3
1.14.13.39
1.14.14.47
4.1.1.19
4.1.1.17
4.3.1.12
1.5.1.1
5.1.1.8
1.2.1.88
Nω-Hydroxy-arginine
N-Carbamoyl-putrescine
3.5.3.12
3.5.1.53
2-Oxo-5-aminovalerate
1.5.1.49
5.1.1.4
4-Hydroxyglutamate semialdehyde
2.6.1.1
1.14.13.39
1.14.14.47
3.5.3.11
Agmatine
1.4.3.3
Linatine
cis-4-Hydroxy-D-proline
2.6.1.21
2.6.1.23
L-Arginine-P
Nitric oxide
p-Coumaroyl-CoA
trans-3-Hydroxy-proline
4.2.1.77
1-Pyrroline-2-carboxylate
1.5.99.13
D-Proline
2-Oxo-4-hydroxy-5-aminovalerate
1.4.3.3
1.5.99.-
(R)-4-Hydroxy-2-oxoglutarate
2.6.1.84
1.4.3.25
2.3.1.64
ACT
p-Coumaroyl-agmatine
p-Coumaroyl-ptrescine
cys-3-Hydroxy-proline
4.2.1.171
1.4.1.12
1.21.4.1
Pyrrole-2-carboxylate
4.1.3.16
1-Pyrroline-4-hydroxy-2-carboxylate
2-Oxoarginine
Feruloyl-CoA
2.3.1.64
ACT
Lysine degradation
5-Amino-pentanoate
Pyruvate
Feruloyl-agmatine
Feruloyl-ptrescine
Tropane, piperidine and pyridine alkaloid biosynthesis
Cysteine and methionine metabolism
3.5.4.22
Glyoxylate
4.1.1.75
1.13.12.1
Putrescine
2,5-Dioxopentanoate
Citrate cycle
PuuA
2.3.1.57
3.5.1.62
L-Aspartate-4-semialdehyde
S-Adenosyl-L-methionine
4-Guanidino-butanal
4-Guanidino-butanamide
γ-L-Glutamyl-putrescine
N-Acetyl-putrescine
Glycine metabolism
Ascorbate and aldarate metabolism
Glycine, serine and threonine metabolism
1.4.3.10
2.6.1.29
1,3-Diamino-propane
4.1.1.50
Pentose and glucuronate interconversions
PuuB
1.4.3.4
1.5.1.43
1.5.1.43
1.5.3.17
Glyoxylate and dicarboxylate metabolism
γ-Glutamyl-γ-amino-butyraldehyde
N4-Acetyl-aminobutanal
1.4.3.22
Carboxy-norspermidine
Carboxy-spermidine
S-Adenosyl-methioninamine
2.6.1.82
2.6.1.113
PuuC
1.2.1.3
4.1.1.96
4.1.1.96
1.2.1.54
3.5.1.4
2.5.1.16
γ-Glutamyl-γ-amino-butyrate
N4-Aceyl-amino-butanoate
4-Amino-butanal
Norspermidine
1.5.3.14
2.5.1.16
Spermine
β-Alanine metabolism
Spermidine
1.5.99.6
2.5.1.22
PuuD
3.5.1.63
1.2.1.3
Polyamine Pathway
1.5.3.16
1.2.1.19
1.2.1.-
1.5.3.17
Glutathione metabolism
ADH pathway
ADC pathway
4-Amino-butanoate
6.3.2.11
3.4.13.18
L-Homocarnosine
4-Guanidino-butanoate
3.5.3.7
3.4.13.20
2.1.4.1
Alanine, aspartate and glutamate metabolism
Butanoate metabolism
Data on KEGG graph
Rendered by Pathview

J)

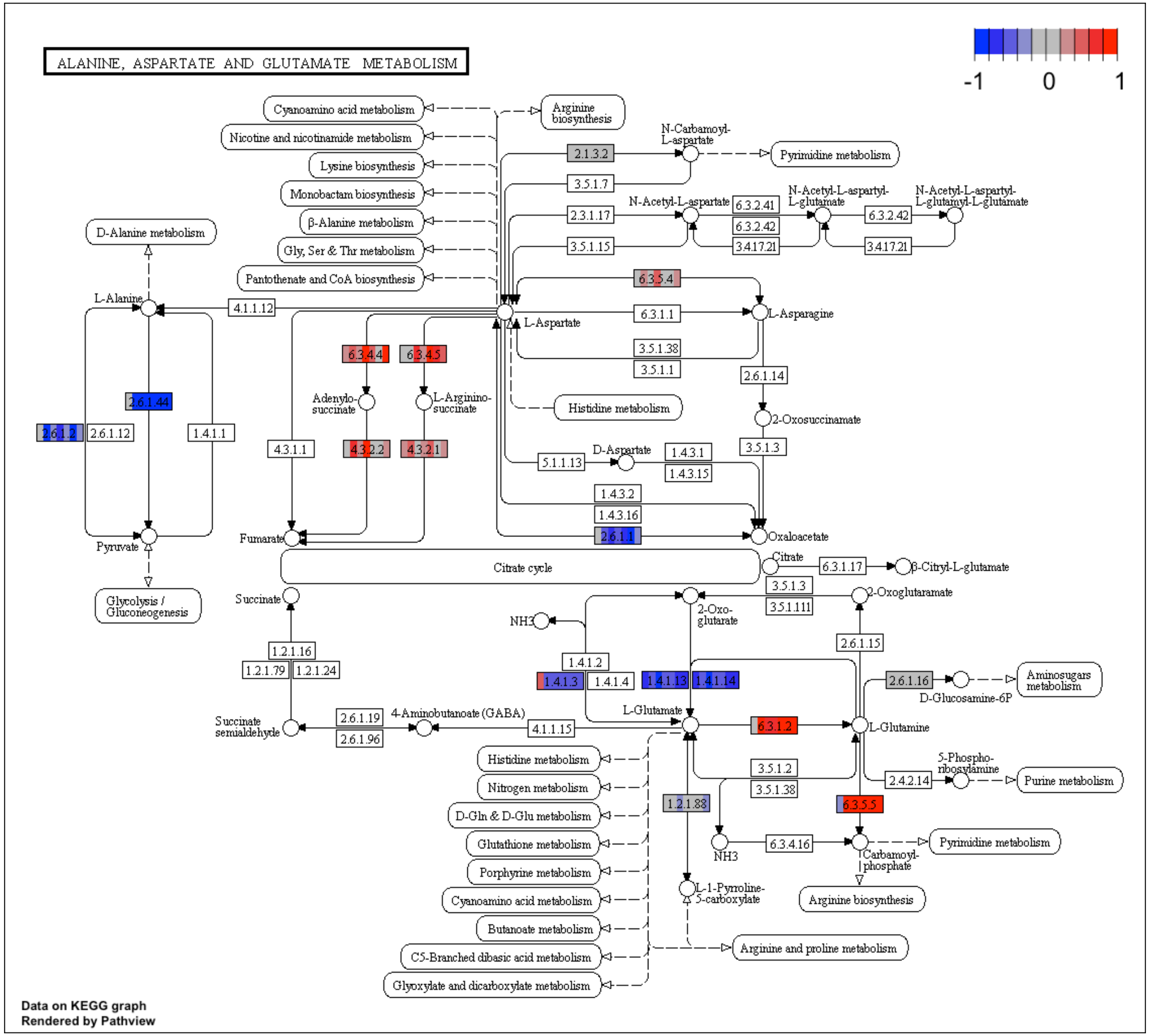

ALANINE, ASPARTATE AND GLUTAMATE METABOLISM
-1
0
1
Cyanoamino acid metabolism
Nicotine and nicotinamide metabolism
Lysine biosynthesis
Monobactam biosynthesis
β-Alanine metabolism
Gly, Ser & Thr metabolism
Pantothenate and CoA biosynthesis
Arginine biosynthesis
N-Carbamoyl-L-aspartate
Pyrimidine metabolism
2.1.3.2
3.5.1.7
2.3.1.17
3.5.1.15
N-Acetyl-L-aspartate
6.3.2.41
6.3.2.42
3.4.17.21
N-Acetyl-L-aspartyl-L-glutamate
N-Acetyl-L-aspartyl-L-glutamyl-L-glutamate
D-Alanine metabolism
L-Alanine
4.1.1.12
6.3.5.4
L-Aspartate
6.3.1.1
L-Asparagine
3.5.1.38
3.5.1.1
6.3.4.4
6.3.4.5
2.6.1.44
2.6.1.2
2.6.1.12
1.4.1.1
Adenylo-succinate
L-Argininosuccinate
Histidine metabolism
2.6.1.14
2-Oxosuccinamate
4.3.1.1
4.3.2.2
4.3.2.1
5.1.1.13
D-Aspartate
1.4.3.1
1.4.3.15
3.5.1.3
1.4.3.2
1.4.3.16
2.6.1.1
Pyruvate
Fumarate
Oxaloacetate
Glycolysis / Gluconeogenesis
Citrate cycle
Citrate
6.3.1.17
β-Citryl-L-glutamate
Succinate
3.5.1.3
3.5.1.111
2-Oxoglutaramate
NH3
2-Oxo-glutarate
1.2.1.16
1.2.1.79
1.2.1.24
1.4.1.2
1.4.1.3
1.4.1.4
1.4.1.13
1.4.1.14
2.6.1.15
2.6.1.16
Aminosugars metabolism
D-Glucosamine-6P
Succinate semialdehyde
2.6.1.19
2.6.1.96
4-Aminobutanoate (GABA)
4.1.1.15
L-Glutamate
6.3.1.2
L-Glutamine
Histidine metabolism
Nitrogen metabolism
D-Gln & D-Glu metabolism
Glutathione metabolism
Porphyrine metabolism
Cyanoamino acid metabolism
Butanoate metabolism
C5-Branched dibasic acid metabolism
Glyoxylate and dicarboxylate metabolism
3.5.1.2
3.5.1.38
5-Phospho-ribosylamine
2.4.2.14
Purine metabolism
1.2.1.88
6.3.5.5
NH3
6.3.4.16
Carbamoyl-phosphate
Pyrimidine metabolism
L-1-Pyrroline-5-carboxylate
Arginine biosynthesis
Arginine and proline metabolism
Data on KEGG graph
Rendered by Pathview

K)

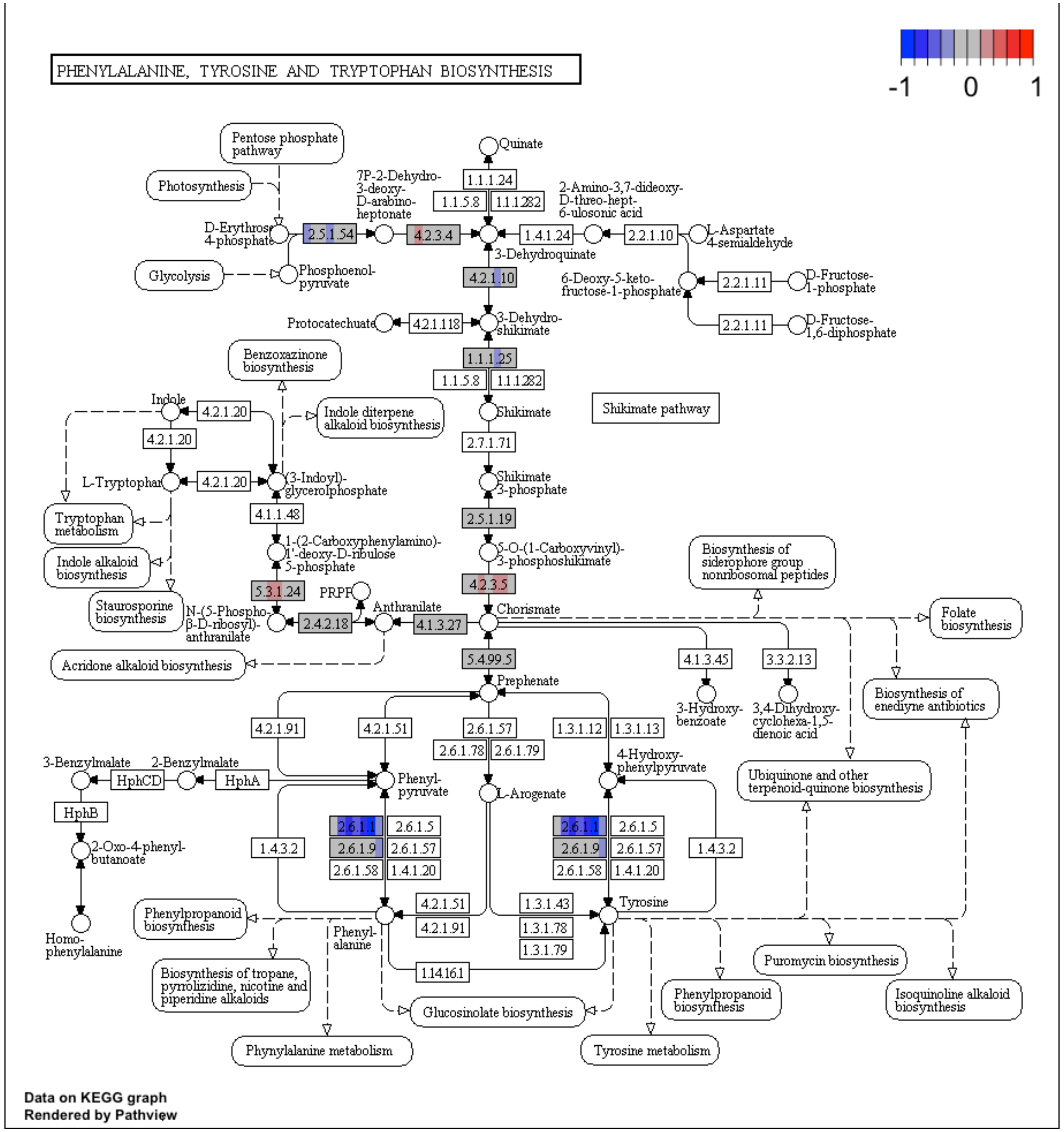
PHENYLALANINE, TYROSINE AND TRYPTOPHAN BIOSYNTHESIS
-1
0
1
Pentose phosphate pathway
Photosynthesis
Glycolysis
D-Erythrose 4-phosphate
Phosphoenol-pyruvate
2.5.1.54
4.2.3.4
7P-2-Dehydro-3-deoxy-D-arabino-heptonate
Quinate
1.1.1.24
1.1.5.8
1.1.1.282
2-Amino-3,7-dideoxy-D-threo-hept-6-ulosonic acid
1.4.1.24
2.2.1.10
L-Aspartate 4-semialdehyde
3-Dehydroquinate
4.2.1.10
6-Deoxy-5-keto-fructose-1-phosphate
2.2.1.11
D-Fructose-1-phosphate
D-Fructose-1,6-diphosphate
Protocatechuate
4.2.1.118
3-Dehydro-shikimate
1.1.1.25
Benzoxazinone biosynthesis
Shikimate
Shikimate pathway
Indole
4.2.1.20
Indole diterpene alkaloid biosynthesis
2.7.1.71
L-Tryptophan
(3-Indoyl)-glycerolphosphate
Shikimate 3-phosphate
4.1.1.48
2.5.1.19
Tryptophan metabolism
Indole alkaloid biosynthesis
1-(2-Carboxyphenylamino)-1'-deoxy-D-ribulose 5-phosphate
5-O-(1-Carboxyvinyl)-3-phosphoshikimate
Biosynthesis of siderophore group nonribosomal peptides
5.3.1.24
PRPP
4.2.3.5
Staurosporine biosynthesis
N-(5-Phospho-β-D-ribosyl)-anthranilate
2.4.2.18
Anthranilate
4.1.3.27
Chorismate
Folate biosynthesis
Acridone alkaloid biosynthesis
5.4.99.5
4.1.3.45
3.3.2.13
Prephenate
3-Hydroxy-benzoate
3,4-Dihydroxy-cyclohexa-1,5-dienoic acid
Biosynthesis of enediyne antibiotics
4.2.1.91
4.2.1.51
2.6.1.57
2.6.1.78
2.6.1.79
1.3.1.12
1.3.1.13
3-Benzylmalate
2-Benzylmalate
HphCD
HphA
Phenyl-pyruvate
4-Hydroxy-phenylpyruvate
L-Arogenate
Ubiquinone and other terpenoid-quinone biosynthesis
HphB
2.6.1.1
2.6.1.5
2.6.1.9
2.6.1.58
1.4.1.20
1.4.3.2
2-Oxo-4-phenyl-butanoate
Homo-phenylalanine
Phenylpropanoid biosynthesis
Phenyl-alanine
1.3.1.43
1.3.1.78
1.3.1.79
Tyrosine
1.14.16.1
Biosynthesis of tropane, pyrrolizidine, nicotine and piperidine alkaloids
Glucosinolate biosynthesis
Puromycin biosynthesis
Isoquinoline alkaloid biosynthesis
Phynylalanine metabolism
Tyrosine metabolism
Data on KEGG graph
Rendered by Pathview

L)

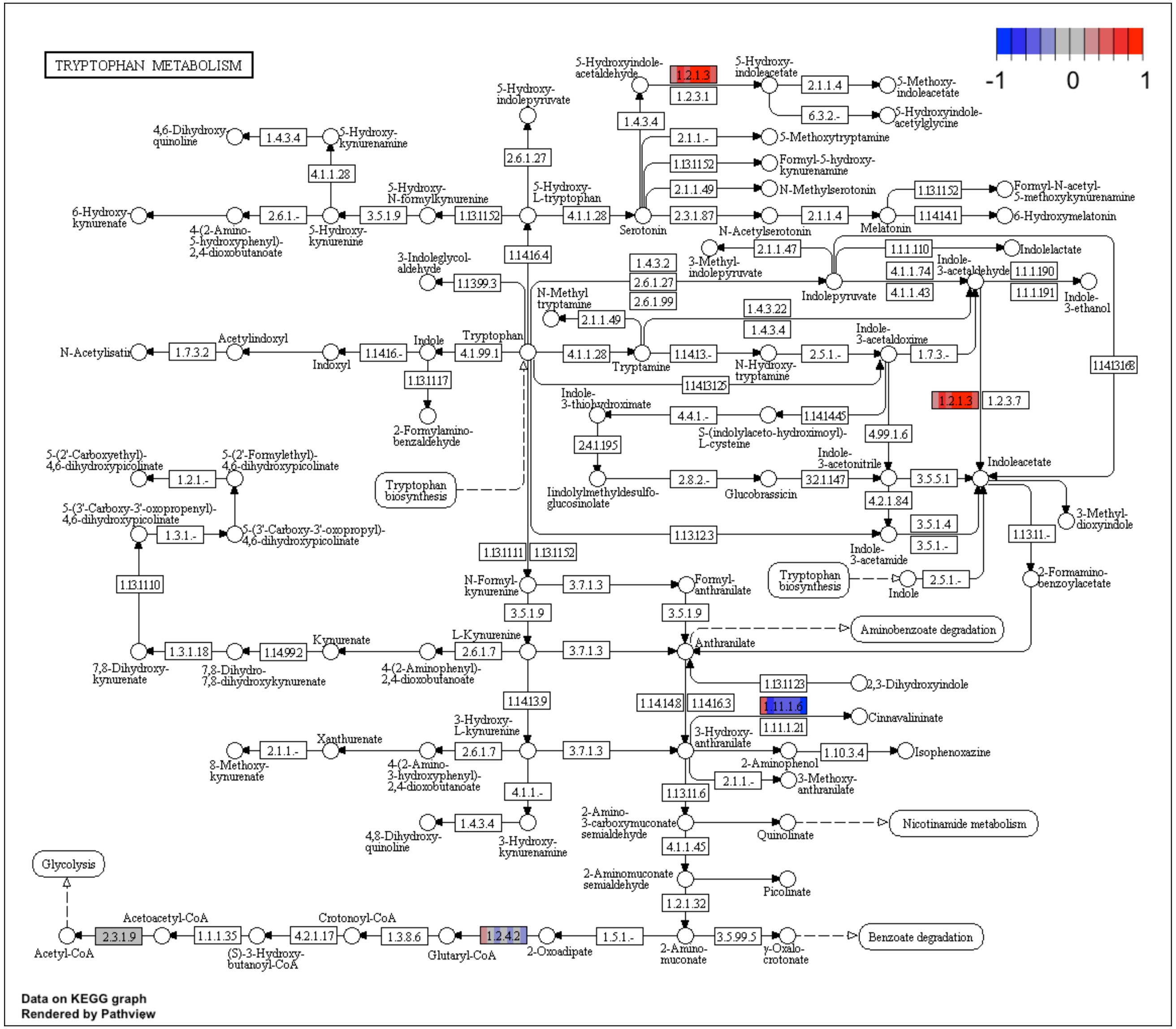
TRYPTOPHAN METABOLISM
-1
0
1
5-Hydroxyindole-acetaldehyde
1.2.1.3
1.2.3.1
5-Hydroxy-indoleacetate
2.1.1.4
5-Methoxy-indoleacetate
6.3.2.-
5-Hydroxyindole-acetylglycine
5-Hydroxy-indolepyruvate
1.4.3.4
2.1.1.-
5-Methoxytryptamine
1.13.11.52
Formyl-5-hydroxy-kynurenamine
2.1.1.49
N-Methylserotonin
1.13.11.52
Formyl-N-acetyl-5-methoxykynurenamine
2.3.1.87
2.1.1.4
1.14.14.1
6-Hydroxymelatonin
Melatonin
N-Acetylserotonin
Serotonin
4,6-Dihydroxy-quinoline
1.4.3.4
5-Hydroxy-kynurenamine
4.1.1.28
2.6.1.27
5-Hydroxy-N-formylkynurenine
5-Hydroxy-L-tryptophan
6-Hydroxy-kynurenate
2.6.1.-
4-(2-Amino-5-hydroxyphenyl)-2,4-dioxobutanoate
3.5.1.9
5-Hydroxy-kynurenine
1.13.11.52
4.1.1.28
1.14.16.4
2.1.1.47
3-Indoleglycol-aldehyde
1.13.99.3
1.4.3.2
2.6.1.27
2.6.1.99
3-Methyl-indolepyruvate
Indolepyruvate
1.1.1.110
Indolelactate
4.1.1.74
Indole-3-acetaldehyde
1.1.1.190
4.1.1.43
1.1.1.191
Indole-3-ethanol
N-Methyl tryptamine
2.1.1.49
1.4.3.22
1.4.3.4
N-Acetylisatin
1.7.3.2
Acetylindoxyl
Indoxyl
1.14.16.-
Indole
4.1.99.1
Tryptophan
4.1.1.28
Tryptamine
1.14.13.-
N-Hydroxy-tryptamine
2.5.1.-
Indole-3-acetaldoxime
1.7.3.-
1.13.11.17
1.14.13.125
1.14.13.168
2-Formylamino-benzaldehyde
Indole-3-thiohydroximate
4.4.1.-
S-(indolylaceto-hydroximoyl)-L-cysteine
1.14.14.45
1.2.1.3
1.2.3.7
2.4.1.195
4.99.1.6
Indole-3-acetonitrile
Indoleacetate
5-(2'-Carboxyethyl)-4,6-dihydroxypicolinate
1.2.1.-
5-(2'-Formylethyl)-4,6-dihydroxypicolinate
Tryptophan biosynthesis
Indolylmethyldesulfo-glucosinolate
2.8.2.-
Glucobrassicin
3.2.1.147
3.5.5.1
4.2.1.84
3-Methyl-dioxyindole
5-(3'-Carboxy-3'-oxopropenyl)-4,6-dihydroxypicolinate
1.3.1.-
5-(3'-Carboxy-3'-oxopropyl)-4,6-dihydroxypicolinate
1.13.12.3
3.5.1.4
1.13.11.-
3.5.1.-
Indole-3-acetamide
1.13.11.11
1.13.11.52
1.13.11.10
N-Formyl-kynurenine
3.7.1.3
Formyl-anthranilate
Tryptophan biosynthesis
Indole
2.5.1.-
2-Formamino-benzoylacetate
3.5.1.9
3.5.1.9
Aminobenzoate degradation
Anthranilate
7,8-Dihydroxy-kynurenate
1.3.1.18
7,8-Dihydro-7,8-dihydroxykynurenate
1.14.99.2
Kynurenate
4-(2-Aminophenyl)-2,4-dioxobutanoate
2.6.1.7
L-Kynurenine
3.7.1.3
1.13.11.23
2,3-Dihydroxyindole
1.14.13.9
1.14.14.8
1.14.16.3
1.11.1.6
Cinnavalininate
1.11.1.21
8-Methoxy-kynurenate
2.1.1.-
Xanthurenate
4-(2-Amino-3-hydroxyphenyl)-2,4-dioxobutanoate
2.6.1.7
3-Hydroxy-L-kynurenine
3.7.1.3
3-Hydroxy-anthranilate
1.10.3.4
Isophenoxazine
2-Aminophenol
2.1.1.-
3-Methoxy-anthranilate
4.1.1.-
1.13.11.6
4,8-Dihydroxy-quinoline
1.4.3.4
3-Hydroxy-kynurenamine
2-Amino-3-carboxymuconate semialdehyde
Quinolinate
Nicotinamide metabolism
4.1.1.45
Glycolysis
2-Aminomuconate semialdehyde
Picolinate
1.2.1.32
Acetyl-CoA
2.3.1.9
Acetoacetyl-CoA
1.1.1.35
(S)-3-Hydroxy-butanoyl-CoA
4.2.1.17
Crotonoyl-CoA
1.3.8.6
Glutaryl-CoA
1.2.4.2
2-Oxoadipate
1.5.1.-
2-Amino-muconate
3.5.99.5
γ-Oxalo-crotonate
Benzoate degradation
Data on KEGG graph
Rendered by Pathview

M)

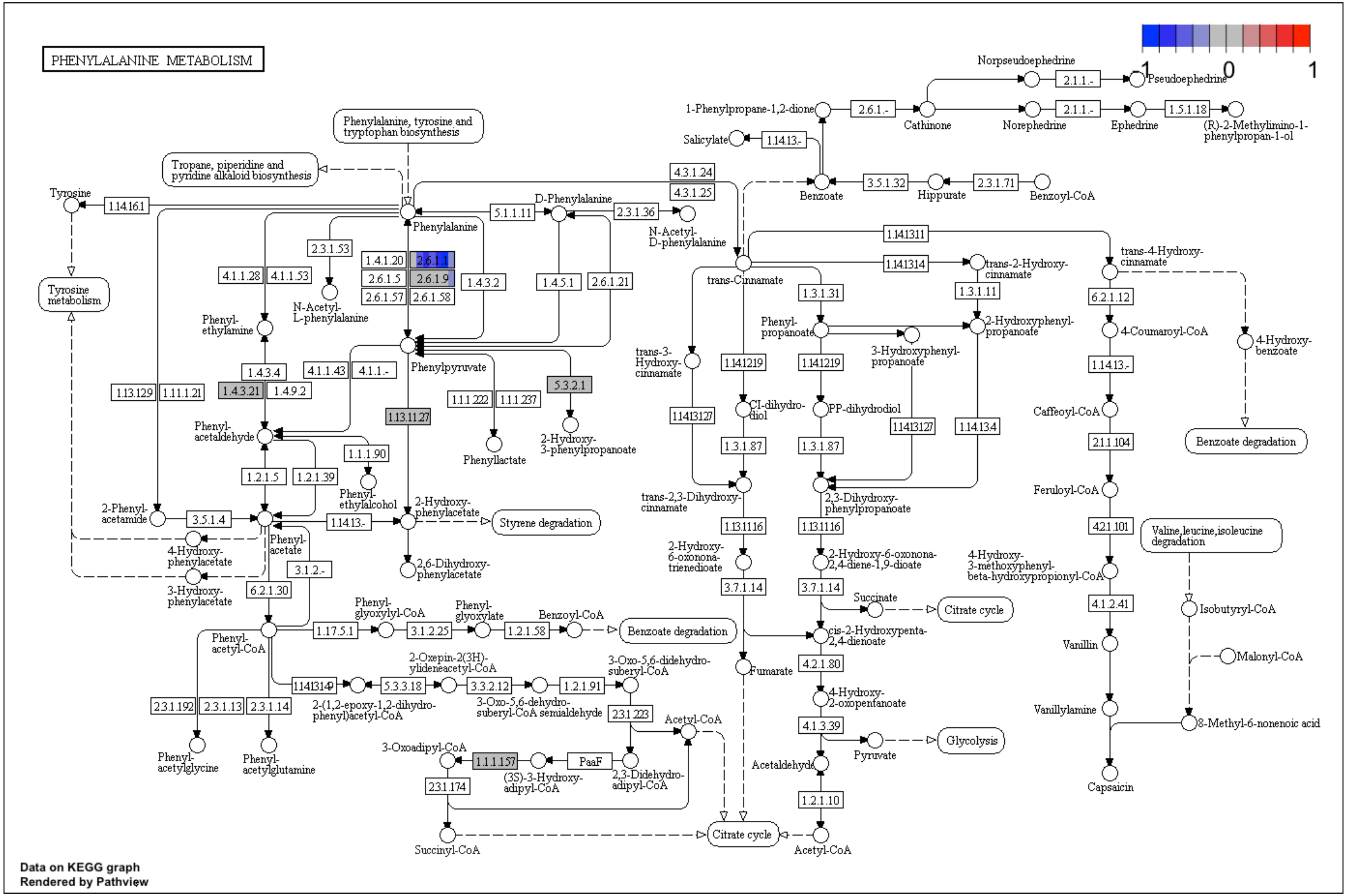

PHENYLALANINE METABOLISM
-1
0
1
Phenylalanine, tyrosine and tryptophan biosynthesis
Tropane, piperidine and pyridine alkaloid biosynthesis
Tyrosine
Tyrosine metabolism
Phenylalanine
D-Phenylalanine
N-Acetyl-D-phenylalanine
N-Acetyl-L-phenylalanine
Phenylethylamine
Phenylpyruvate
Phenylacetaldehyde
Phenyllactate
2-Hydroxy-3-phenylpropanoate
Phenylethylalcohol
2-Phenylacetamide
Phenylacetate
4-Hydroxyphenylacetate
3-Hydroxyphenylacetate
2-Hydroxyphenylacetate
2,6-Dihydroxyphenylacetate
Styrene degradation
Phenylacetyl-CoA
Phenylglyoxylyl-CoA
Phenylglyoxylate
Benzoyl-CoA
Benzoate degradation
Phenylacetylglycine
Phenylacetylglutamine
2-Oxepin-2(3H)-ylideneacetyl-CoA
2-(1,2-epoxy-1,2-dihydrophenyl)acetyl-CoA
3-Oxo-5,6-dehydrosuberyl-CoA semialdehyde
3-Oxo-5,6-didehydrosuberyl-CoA
3-Oxoadipyl-CoA
(3S)-3-Hydroxyadipyl-CoA
2,3-Didehydroadipyl-CoA
Acetyl-CoA
Succinyl-CoA
Citrate cycle
1-Phenylpropane-1,2-dione
Salicylate
Cathinone
Norpseudoephedrine
Pseudoephedrine
Norephedrine
Ephedrine
(R)-2-Methylimino-1-phenylpropan-1-ol
Benzoate
Hippurate
Benzoyl-CoA
trans-Cinnamate
trans-2-Hydroxycinnamate
trans-4-Hydroxycinnamate
trans-3-Hydroxycinnamate
Phenylpropanoate
3-Hydroxyphenylpropanoate
2-Hydroxyphenylpropanoate
CI-dihydrodiol
PP-dihydrodiol
trans-2,3-Dihydroxycinnamate
2,3-Dihydroxyphenylpropanoate
2-Hydroxy-6-oxonona-trienedioate
2-Hydroxy-6-oxonona-2,4-diene-1,9-dioate
Succinate
Fumarate
cis-2-Hydroxypenta-2,4-dienoate
4-Hydroxy-2-oxopentanoate
Pyruvate
Glycolysis
Acetaldehyde
Acetyl-CoA
4-Coumaroyl-CoA
4-Hydroxybenzoate
Benzoate degradation
Caffeoyl-CoA
Feruloyl-CoA
4-Hydroxy-3-methoxyphenyl-beta-hydroxypropionyl-CoA
Vanillin
Vanillylamine
Capsaicin
Valine, leucine, isoleucine degradation
Isobutyryl-CoA
Malonyl-CoA
8-Methyl-6-nonenoic acid
1.14.16.1
5.1.1.11
2.3.1.36
2.3.1.53
1.4.1.20
2.6.1.1
2.6.1.5
2.6.1.9
2.6.1.57
2.6.1.58
1.4.3.2
1.4.5.1
2.6.1.21
4.1.1.28
4.1.1.53
1.4.3.4
1.4.3.21
1.4.9.2
4.1.1.43
4.1.1.-
1.13.12.9
1.11.1.21
1.13.11.27
1.1.1.222
1.1.1.237
5.3.2.1
1.1.1.90
1.2.1.5
1.2.1.39
3.5.1.4
1.14.13.-
3.1.2.-
6.2.1.30
1.17.5.1
3.1.2.25
1.2.1.58
1.14.13.149
5.3.3.18
3.3.2.12
1.2.1.91
2.3.1.192
2.3.1.13
2.3.1.14
2.3.1.223
2.3.1.174
1.1.1.157
PaaF
4.3.1.24
4.3.1.25
2.6.1.-
1.14.13.-
2.1.1.-
1.5.1.18
3.5.1.32
2.3.1.71
1.14.13.11
1.14.13.14
1.3.1.31
1.3.1.11
1.14.12.19
1.14.13.127
1.3.1.87
1.14.13.4
1.13.11.16
3.7.1.14
4.2.1.80
4.1.3.39
1.2.1.10
6.2.1.12
2.1.1.104
4.2.1.101
4.1.2.41
Data on KEGG graph
Rendered by Pathview

N)

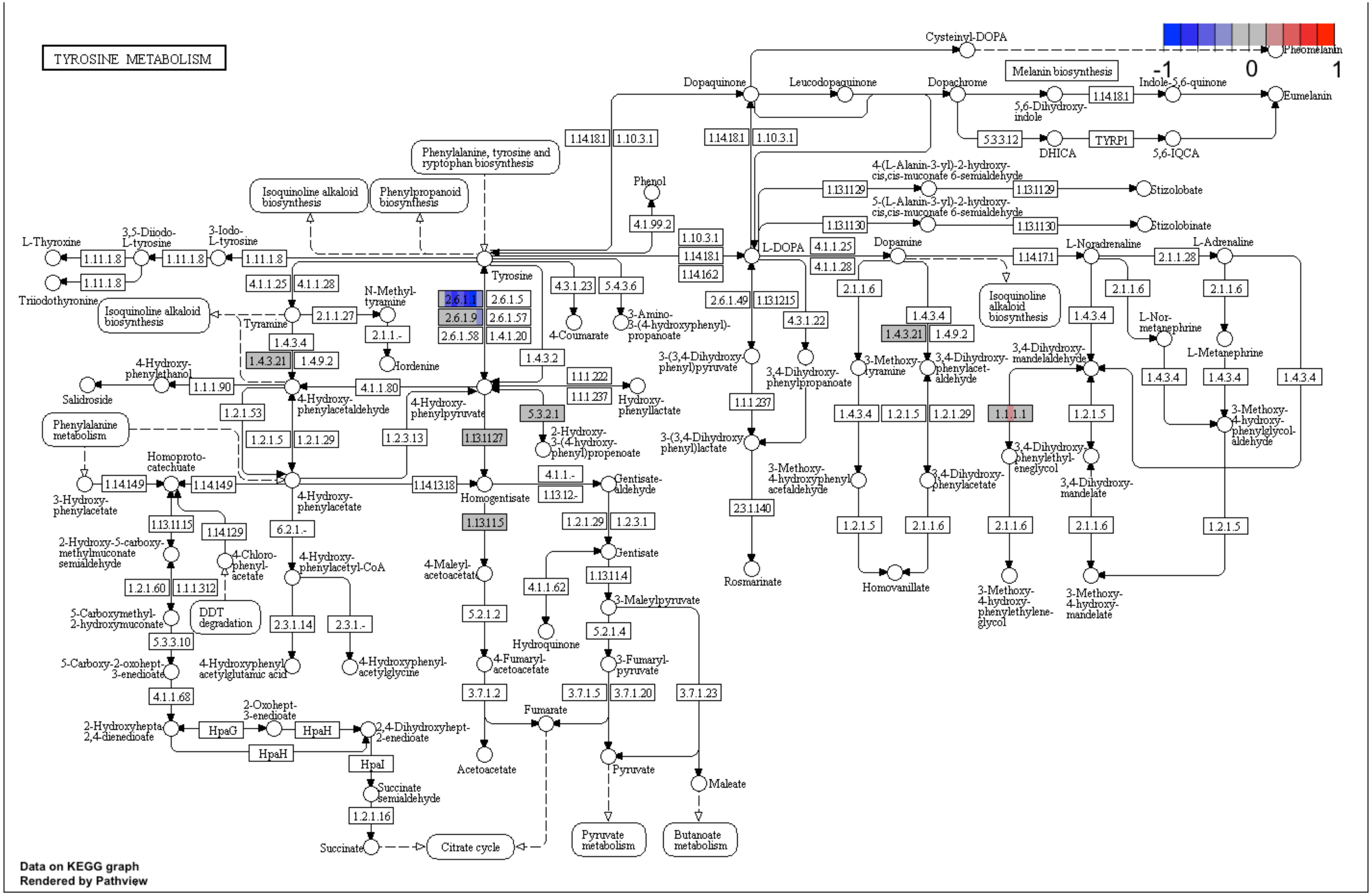
TYROSINE METABOLISM
-1
0
1
Cysteinyl-DOPA
Pheomelanin
Melanin biosynthesis
Dopaquinone
Leucodopaquinone
Dopachrome
5,6-Dihydroxy-indole
Indole-5,6-quinone
Eumelanin
DHICA
5,6-IQCA
TYRP1
Phenylalanine, tyrosine and ryptophan biosynthesis
Isoquinoline alkaloid biosynthesis
Phenylpropanoid biosynthesis
Phenol
L-Thyroxine
3,5-Diiodo-L-tyrosine
3-Iodo-L-tyrosine
Triiodothyronine
Tyrosine
L-DOPA
Dopamine
L-Noradrenaline
L-Adrenaline
4-(L-Alanin-3-yl)-2-hydroxy-cis,cis-muconate 6-semialdehyde
5-(L-Alanin-3-yl)-2-hydroxy-cis,cis-muconate 6-semialdehyde
Stizolobate
Stizolobinate
N-Methyl-tyramine
Tyramine
Hordenine
4-Coumarate
3-Amino-3-(4-hydroxyphenyl)-propanoate
3-(3,4-Dihydroxy-phenyl)pyruvate
3,4-Dihydroxy-phenylpropanoate
3-(3,4-Dihydroxy-phenyl)lactate
Rosmarinate
3-Methoxy-tyramine
3,4-Dihydroxy-phenylacet-aldehyde
3,4-Dihydroxy-mandelaldehyde
L-Nor-metanephrine
L-Metanephrine
3-Methoxy-4-hydroxy-phenylglycol-aldehyde
3-Methoxy-4-hydroxyphenyl-acetaldehyde
3,4-Dihydroxy-phenylacetate
3,4-Dihydroxy-phenylethyl-eneglycol
3,4-Dihydroxy-mandelate
Homovanillate
3-Methoxy-4-hydroxy-phenylethylene-glycol
3-Methoxy-4-hydroxy-mandelate
Salidroside
4-Hydroxy-phenylethanol
4-Hydroxy-phenylacetaldehyde
4-Hydroxy-phenylpyruvate
Hydroxy-phenyllactate
2-Hydroxy-3-(4-hydroxy-phenyl)propenoate
Phenylalanine metabolism
3-Hydroxy-phenylacetate
Homoproto-catechuate
4-Hydroxy-phenylacetate
Homogentisate
Gentisate-aldehyde
Gentisate
2-Hydroxy-5-carboxy-methylmuconate semialdehyde
4-Chloro-phenyl-acetate
DDT degradation
4-Hydroxy-phenylacetyl-CoA
4-Maleyl-acetoacetate
3-Maleylpyruvate
Hydroquinone
5-Carboxymethyl-2-hydroxymuconate
5-Carboxy-2-oxohept-3-enedioate
4-Hydroxyphenyl-acetylglutamic acid
4-Hydroxyphenyl-acetylglycine
4-Fumaryl-acetoacetate
3-Fumaryl-pyruvate
Fumarate
Acetoacetate
Pyruvate
Maleate
2-Hydroxyhepta-2,4-dienedioate
2-Oxohept-3-enedioate
2,4-Dihydroxyhept-2-enedioate
HpaG
HpaH
HpaI
Succinate semialdehyde
Succinate
Citrate cycle
Pyruvate metabolism
Butanoate metabolism
1.14.18.1
1.10.3.1
5.3.3.12
1.13.11.29
1.13.11.30
4.1.99.2
1.14.16.2
4.1.1.25
4.1.1.28
1.14.17.1
2.1.1.28
1.11.1.8
2.6.1.1
2.6.1.5
2.6.1.9
2.6.1.57
2.6.1.58
1.4.1.20
2.1.1.27
2.1.1.-
1.4.3.4
1.4.3.21
1.4.9.2
4.3.1.23
5.4.3.6
2.6.1.49
1.13.12.15
4.3.1.22
2.1.1.6
1.4.3.2
1.1.1.222
1.1.1.237
5.3.2.1
1.1.1.90
4.1.1.80
1.2.1.53
1.2.1.5
1.2.1.29
1.2.3.13
1.13.11.27
1.14.13.18
1.14.14.9
4.1.1.-
1.13.12.-
1.1.1.1
2.3.1.140
1.13.11.15
1.14.12.9
6.2.1.-
1.13.11.5
1.2.3.1
1.2.1.60
1.1.1.312
4.1.1.62
1.13.11.4
5.2.1.2
5.2.1.4
5.3.3.10
2.3.1.14
2.3.1.-
3.7.1.2
3.7.1.5
3.7.1.20
3.7.1.23
4.1.1.68
1.2.1.16
Data on KEGG graph
Rendered by Pathview

O)

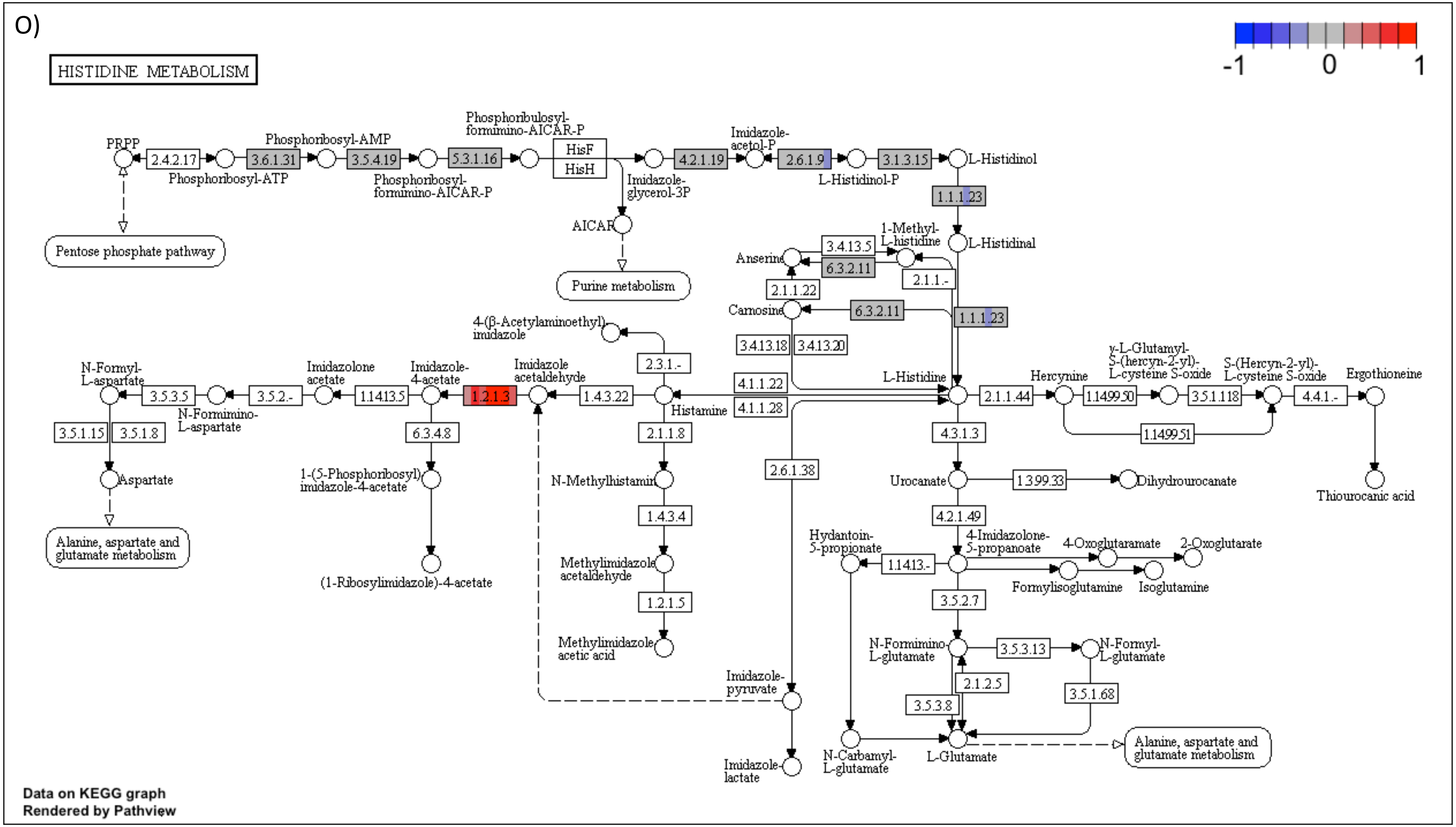

HISTIDINE METABOLISM
-1
0
1
PRPP
2.4.2.17
Phosphoribosyl-ATP
3.6.1.31
Phosphoribosyl-AMP
3.5.4.19
Phosphoribosyl-formimino-AICAR-P
5.3.1.16
Phosphoribulosyl-formimino-AICAR-P
HisF
HisH
AICAR
Imidazole-glycerol-3P
4.2.1.19
Imidazole-acetol-P
2.6.1.9
L-Histidinol-P
3.1.3.15
L-Histidinol
1.1.1.23
L-Histidinal
Pentose phosphate pathway
Purine metabolism
1-Methyl-L-histidine
3.4.13.5
6.3.2.11
Anserine
2.1.1.-
2.1.1.22
Carnosine
6.3.2.11
1.1.1.23
3.4.13.18
3.4.13.20
4-(β-Acetylaminoethyl)imidazole
2.3.1.-
N-Formyl-L-aspartate
3.5.3.5
N-Formimino-L-aspartate
3.5.2.-
Imidazolone acetate
1.14.13.5
Imidazole-4-acetate
1.2.1.3
Imidazole acetaldehyde
1.4.3.22
Histamine
4.1.1.22
4.1.1.28
L-Histidine
2.1.1.44
Hercynine
1.14.99.50
γ-L-Glutamyl-S-(hercyn-2-yl)-L-cysteine S-oxide
3.5.1.118
S-(Hercyn-2-yl)-L-cysteine S-oxide
4.4.1.-
Ergothioneine
1.14.99.51
3.5.1.15
3.5.1.8
Aspartate
6.3.4.8
1-(5-Phosphoribosyl)imidazole-4-acetate
2.1.1.8
N-Methylhistamine
1.4.3.4
Methylimidazole acetaldehyde
1.2.1.5
Methylimidazole acetic acid
2.6.1.38
4.3.1.3
Urocanate
1.3.99.33
Dihydrourocanate
Thiourocanic acid
Alanine, aspartate and glutamate metabolism
(1-Ribosylimidazole)-4-acetate
4.2.1.49
Hydantoin-5-propionate
4-Imidazolone-5-propanoate
1.14.13.-
4-Oxoglutaramate
2-Oxoglutarate
Formylisoglutamine
Isoglutamine
3.5.2.7
N-Formimino-L-glutamate
3.5.3.13
N-Formyl-L-glutamate
2.1.2.5
3.5.3.8
3.5.1.68
Imidazole-pyruvate
Imidazole-lactate
N-Carbamyl-L-glutamate
L-Glutamate
Alanine, aspartate and glutamate metabolism
Data on KEGG graph
Rendered by Pathview

P)

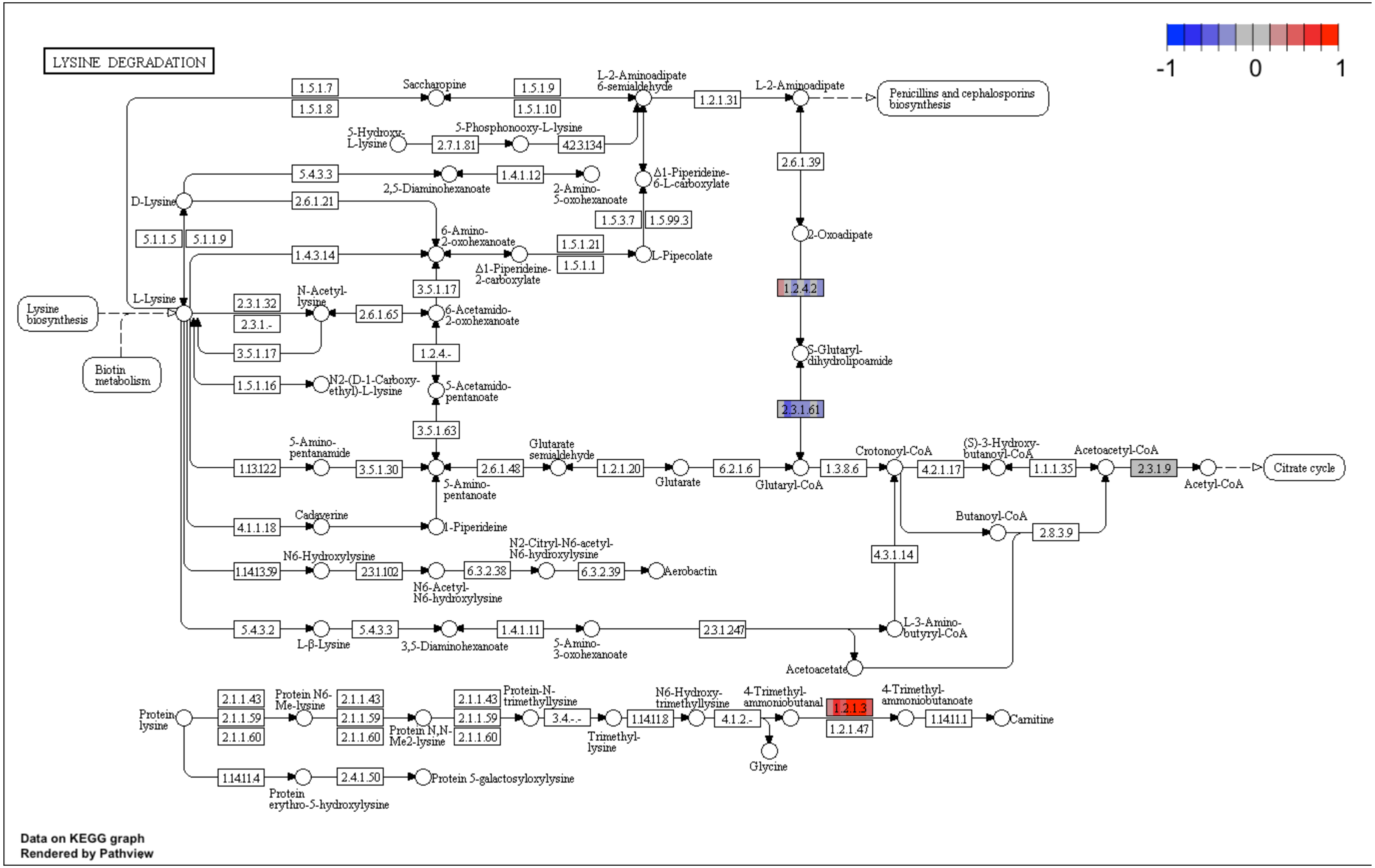
LYSINE DEGRADATION
-1
0
1
1.5.1.7
1.5.1.8
Saccharopine
1.5.1.9
1.5.1.10
L-2-Aminoadipate 6-semialdehyde
1.2.1.31
L-2-Aminoadipate
Penicillins and cephalosporins biosynthesis
5-Hydroxy-L-lysine
2.7.1.81
5-Phosphonooxy-L-lysine
4.2.3.134
5.4.3.3
2,5-Diaminohexanoate
1.4.1.12
2-Amino-5-oxohexanoate
Δ1-Piperideine-6-L-carboxylate
2.6.1.39
D-Lysine
2.6.1.21
1.5.3.7
1.5.99.3
2-Oxoadipate
5.1.1.5
5.1.1.9
6-Amino-2-oxohexanoate
1.4.3.14
Δ1-Piperideine-2-carboxylate
1.5.1.21
1.5.1.1
L-Pipecolate
1.2.4.2
3.5.1.17
L-Lysine
Lysine biosynthesis
2.3.1.32
2.3.1.-
N-Acetyl-lysine
2.6.1.65
6-Acetamido-2-oxohexanoate
3.5.1.17
1.2.4.-
S-Glutaryl-dihydrolipoamide
Biotin metabolism
1.5.1.16
N2-(D-1-Carboxy-ethyl)-L-lysine
5-Acetamido-pentanoate
2.3.1.61
3.5.1.63
5-Amino-pentanamide
Glutarate semialdehyde
1.13.12.2
3.5.1.30
2.6.1.48
1.2.1.20
Glutarate
6.2.1.6
Glutaryl-CoA
1.3.8.6
Crotonoyl-CoA
4.2.1.17
(S)-3-Hydroxy-butanoyl-CoA
1.1.1.35
Acetoacetyl-CoA
2.3.1.9
Acetyl-CoA
Citrate cycle
5-Amino-pentanoate
4.1.1.18
Cadaverine
1-Piperideine
Butanoyl-CoA
2.8.3.9
4.3.1.14
1.14.13.59
N6-Hydroxylysine
2.3.1.102
N6-Acetyl-N6-hydroxylysine
6.3.2.38
N2-Citryl-N6-acetyl-N6-hydroxylysine
6.3.2.39
Aerobactin
5.4.3.2
L-β-Lysine
5.4.3.3
3,5-Diaminohexanoate
1.4.1.11
5-Amino-3-oxohexanoate
2.3.1.247
L-3-Amino-butyryl-CoA
Acetoacetate
Protein lysine
2.1.1.43
2.1.1.59
2.1.1.60
Protein N6-Me-lysine
2.1.1.43
2.1.1.59
2.1.1.60
Protein N,N-Me2-lysine
2.1.1.43
2.1.1.59
2.1.1.60
Protein-N-trimethyllysine
3.4.-.-
Trimethyl-lysine
1.14.11.8
N6-Hydroxy-trimethyllysine
4.1.2.-
Glycine
4-Trimethyl-ammoniobutanal
1.2.1.3
1.2.1.47
4-Trimethyl-ammoniobutanoate
1.14.11.1
Carnitine
1.14.11.4
Protein erythro-5-hydroxylysine
2.4.1.50
Protein 5-galactosyloxylysine
Data on KEGG graph
Rendered by Pathview

Q)

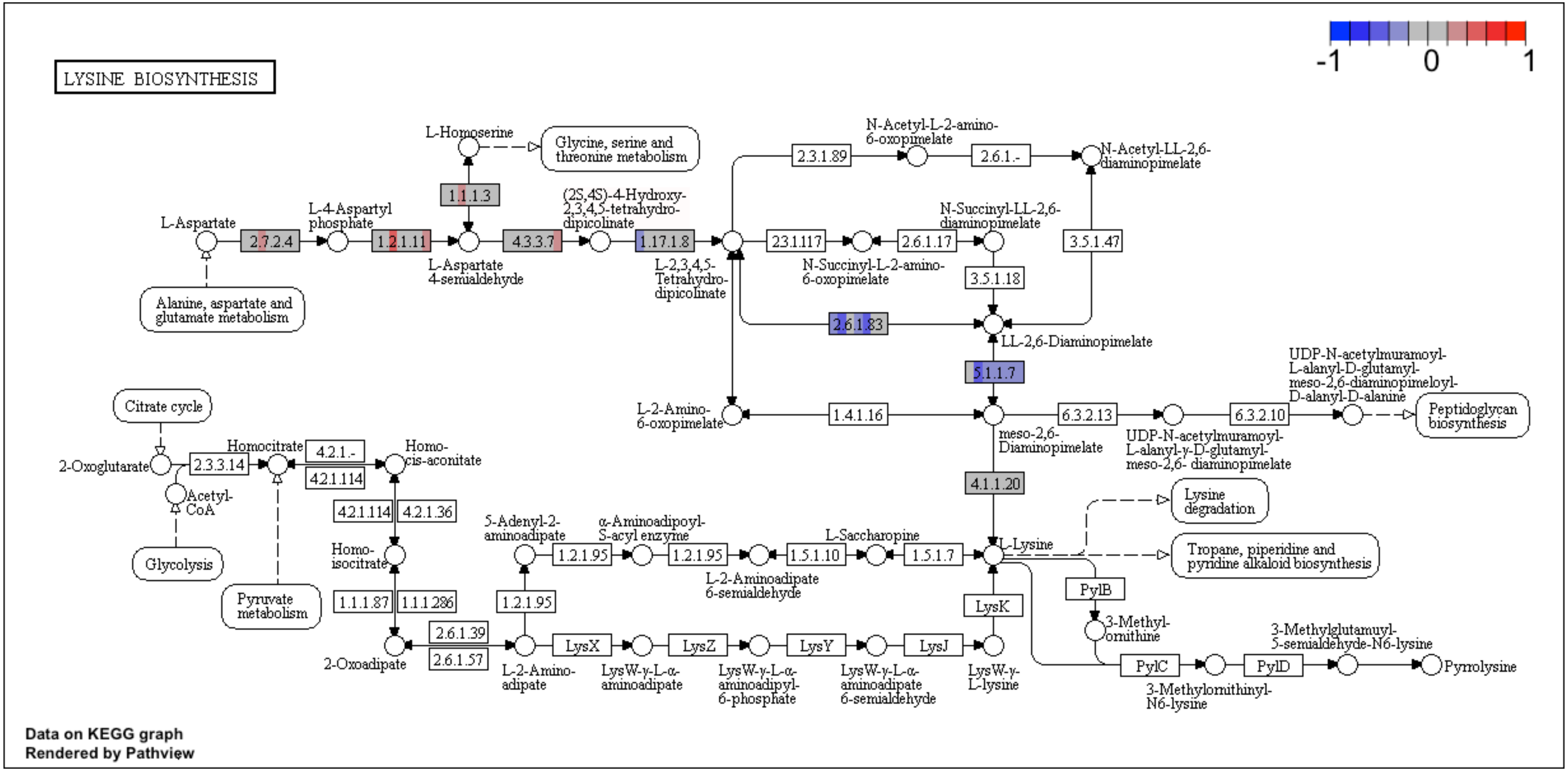

LYSINE BIOSYNTHESIS
-1 0 1
L-Aspartate
2.7.2.4
L-4-Aspartyl phosphate
1.2.1.11
L-Homoserine
1.1.1.3
Glycine, serine and threonine metabolism
L-Aspartate 4-semialdehyde
4.3.3.7
(2S,4S)-4-Hydroxy-2,3,4,5-tetrahydro-dipicolinate
1.17.1.8
L-2,3,4,5-Tetrahydro-dipicolinate
Alanine, aspartate and glutamate metabolism
2.3.1.89
N-Acetyl-L-2-amino-6-oxopimelate
2.6.1.-
N-Acetyl-LL-2,6-diaminopimelate
2.3.1.117
N-Succinyl-L-2-amino-6-oxopimelate
2.6.1.17
N-Succinyl-LL-2,6-diaminopimelate
3.5.1.47
3.5.1.18
2.6.1.83
LL-2,6-Diaminopimelate
5.1.1.7
L-2-Amino-6-oxopimelate
1.4.1.16
meso-2,6-Diaminopimelate
6.3.2.13
UDP-N-acetylmuramoyl-L-alanyl-γ-D-glutamyl-meso-2,6- diaminopimelate
6.3.2.10
UDP-N-acetylmuramoyl-L-alanyl-D-glutamyl-meso-2,6-diaminopimeloyl-D-alanyl-D-alanine
Peptidoglycan biosynthesis
4.1.1.20
Citrate cycle
2-Oxoglutarate
2.3.3.14
Homocitrate
4.2.1.-
4.2.1.114
Homo-cis-aconitate
Acetyl-CoA
Glycolysis
Pyruvate metabolism
4.2.1.114
4.2.1.36
Homo-isocitrate
1.1.1.87
1.1.1.286
2-Oxoadipate
2.6.1.39
2.6.1.57
5-Adenyl-2-aminoadipate
α-Aminoadipoyl-S-acyl enzyme
1.2.1.95
1.2.1.95
1.2.1.95
L-2-Aminoadipate 6-semialdehyde
1.5.1.10
L-Saccharopine
1.5.1.7
L-Lysine
Lysine degradation
Tropane, piperidine and pyridine alkaloid biosynthesis
L-2-Amino-adipate
LysX
LysW-γ-L-α-aminoadipate
LysZ
LysW-γ-L-α-aminoadipyl-6-phosphate
LysY
LysW-γ-L-α-aminoadipate 6-semialdehyde
LysJ
LysW-γ-L-lysine
LysK
PylB
3-Methyl-ornithine
PylC
3-Methylornithinyl-N6-lysine
PylD
3-Methylglutamuyl-5-semialdehyde-N6-lysine
Pyrrolysine
Data on KEGG graph
Rendered by Pathview

R)

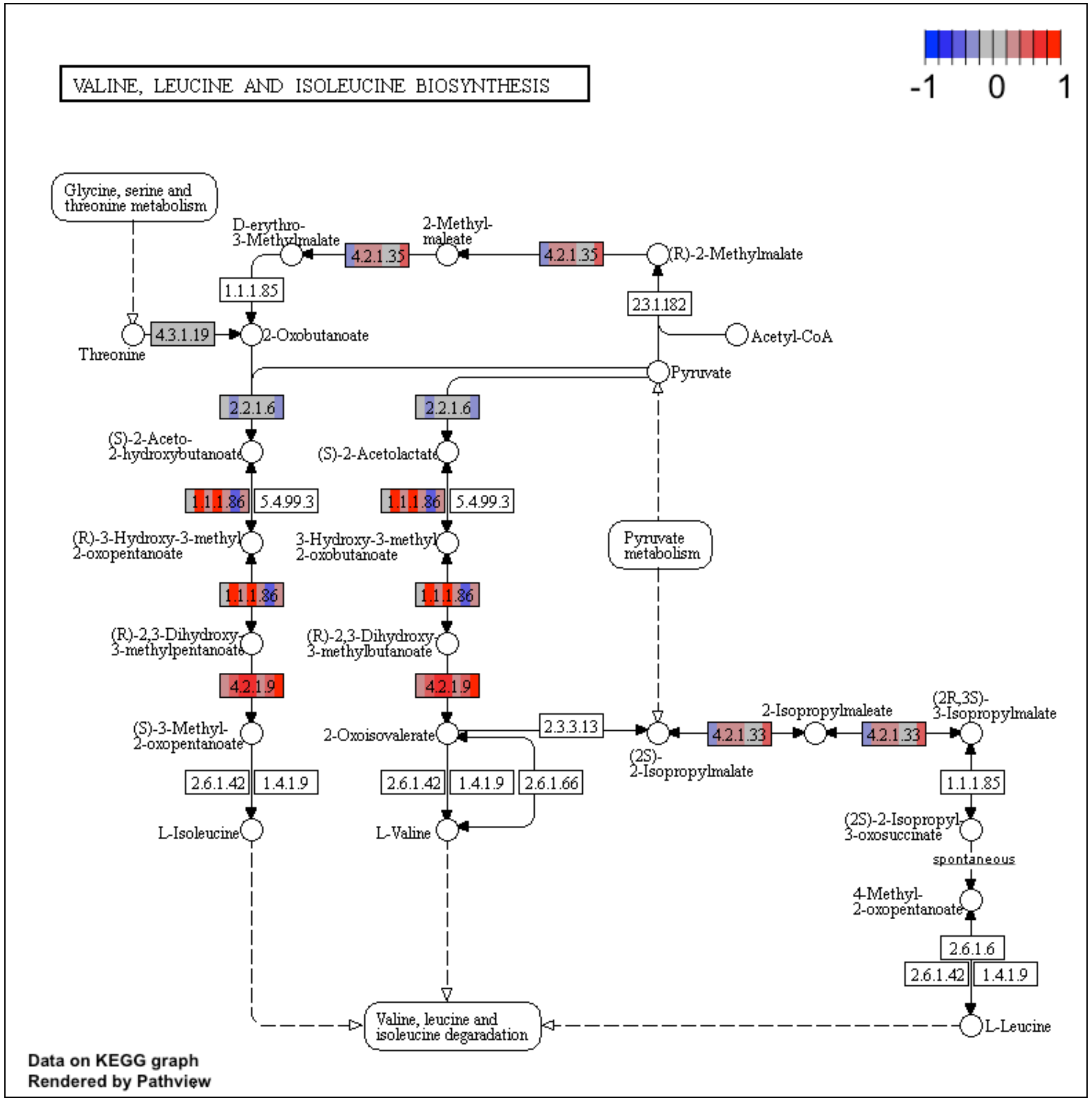

VALINE, LEUCINE AND ISOLEUCINE BIOSYNTHESIS
-1 0 1
Glycine, serine and threonine metabolism
D-erythro-3-Methylmalate
4.2.1.35
2-Methyl-maleate
4.2.1.35
(R)-2-Methylmalate
1.1.1.85
2.3.1.182
Threonine
4.3.1.19
2-Oxobutanoate
Acetyl-CoA
Pyruvate
2.2.1.6
2.2.1.6
(S)-2-Aceto-2-hydroxybutanoate
(S)-2-Acetolactate
1.1.1.86
5.4.99.3
1.1.1.86
5.4.99.3
(R)-3-Hydroxy-3-methyl-2-oxopentanoate
3-Hydroxy-3-methyl-2-oxobutanoate
Pyruvate metabolism
1.1.1.86
1.1.1.86
(R)-2,3-Dihydroxy-3-methylpentanoate
(R)-2,3-Dihydroxy-3-methylbutanoate
4.2.1.9
4.2.1.9
(S)-3-Methyl-2-oxopentanoate
2-Oxoisovalerate
2.3.3.13
2-Isopropylmaleate
(2R,3S)-3-Isopropylmalate
4.2.1.33
4.2.1.33
(2S)-2-Isopropylmalate
2.6.1.42
1.4.1.9
2.6.1.42
1.4.1.9
2.6.1.66
1.1.1.85
L-Isoleucine
L-Valine
(2S)-2-Isopropyl-3-oxosuccinate
spontaneous
4-Methyl-2-oxopentanoate
2.6.1.6
2.6.1.42
1.4.1.9
Valine, leucine and isoleucine degaradation
L-Leucine
Data on KEGG graph
Rendered by Pathview

S)

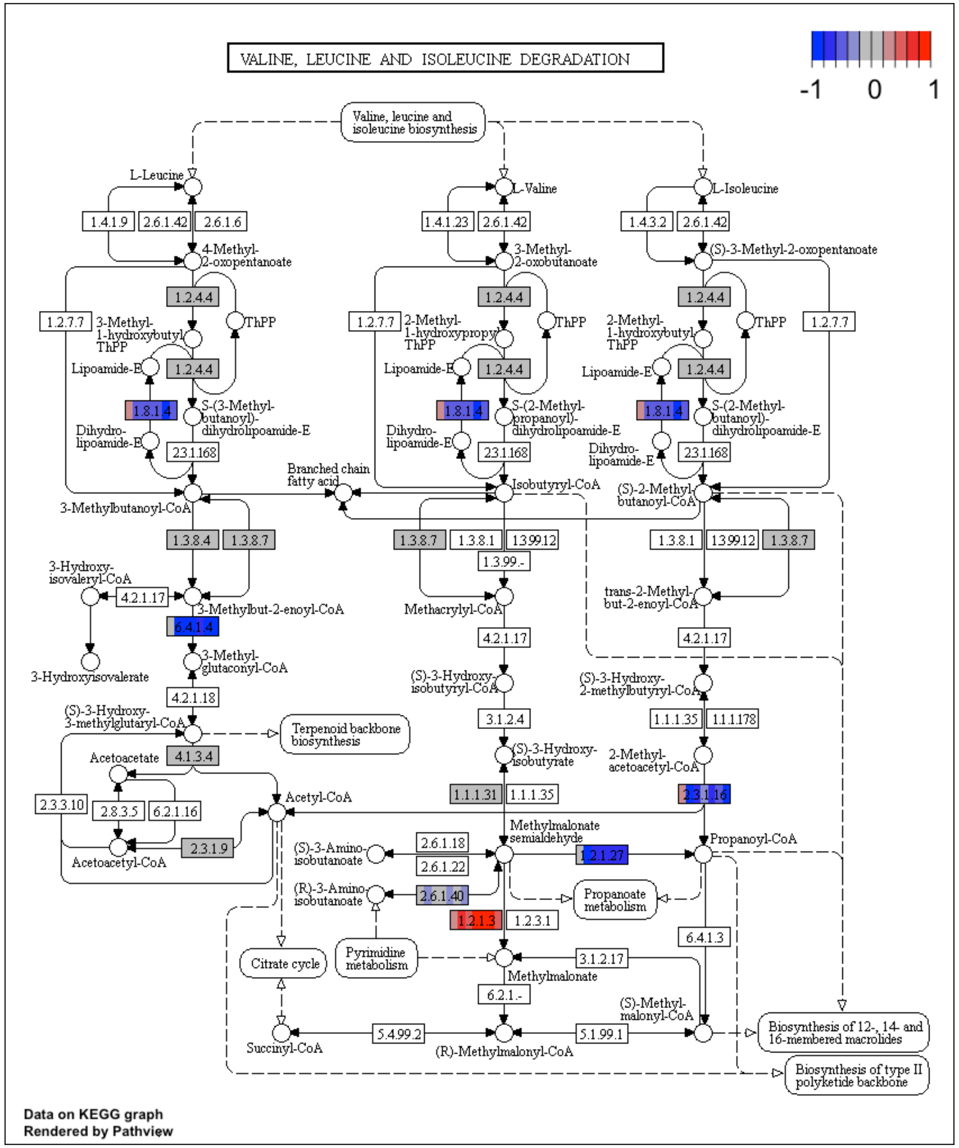
VALINE, LEUCINE AND ISOLEUCINE DEGRADATION
-1
0
1
Valine, leucine and isoleucine biosynthesis
L-Leucine
L-Valine
L-Isoleucine
1.4.1.9
2.6.1.42
2.6.1.6
1.4.1.23
1.4.3.2
4-Methyl-2-oxopentanoate
3-Methyl-2-oxobutanoate
(S)-3-Methyl-2-oxopentanoate
1.2.4.4
1.2.7.7
3-Methyl-1-hydroxybutyl-ThPP
2-Methyl-1-hydroxypropyl-ThPP
2-Methyl-1-hydroxybutyl-ThPP
ThPP
Lipoamide-E
1.8.1.4
S-(3-Methylbutanoyl)-dihydrolipoamide-E
S-(2-Methylpropanoyl)-dihydrolipoamide-E
S-(2-Methylbutanoyl)-dihydrolipoamide-E
Dihydrolipoamide-E
2.3.1.168
Branched chain fatty acid
3-Methylbutanoyl-CoA
Isobutyryl-CoA
(S)-2-Methylbutanoyl-CoA
1.3.8.4
1.3.8.7
1.3.8.1
1.3.99.12
1.3.99.-
3-Hydroxyisovaleryl-CoA
4.2.1.17
3-Methylbut-2-enoyl-CoA
Methacrylyl-CoA
trans-2-Methylbut-2-enoyl-CoA
6.4.1.4
3-Hydroxyisovalerate
3-Methylglutaconyl-CoA
(S)-3-Hydroxyisobutyryl-CoA
(S)-3-Hydroxy-2-methylbutyryl-CoA
4.2.1.18
3.1.2.4
1.1.1.35
1.1.1.178
(S)-3-Hydroxy-3-methylglutaryl-CoA
Terpenoid backbone biosynthesis
(S)-3-Hydroxyisobutyrate
2-Methylacetoacetyl-CoA
Acetoacetate
4.1.3.4
2.3.3.10
2.8.3.5
6.2.1.16
Acetyl-CoA
1.1.1.31
2.3.1.16
Methylmalonate semialdehyde
Propanoyl-CoA
Acetoacetyl-CoA
2.3.1.9
(S)-3-Aminoisobutanoate
2.6.1.18
2.6.1.22
1.2.1.27
(R)-3-Aminoisobutanoate
2.6.1.40
Propanoate metabolism
1.2.1.3
1.2.3.1
6.4.1.3
Citrate cycle
Pyrimidine metabolism
3.1.2.17
Methylmalonate
6.2.1.-
(S)-Methylmalonyl-CoA
Biosynthesis of 12-, 14- and 16-membered macrolides
Succinyl-CoA
5.4.99.2
(R)-Methylmalonyl-CoA
5.1.99.1
Biosynthesis of type II polyketide backbone
Data on KEGG graph
Rendered by Pathview

T)

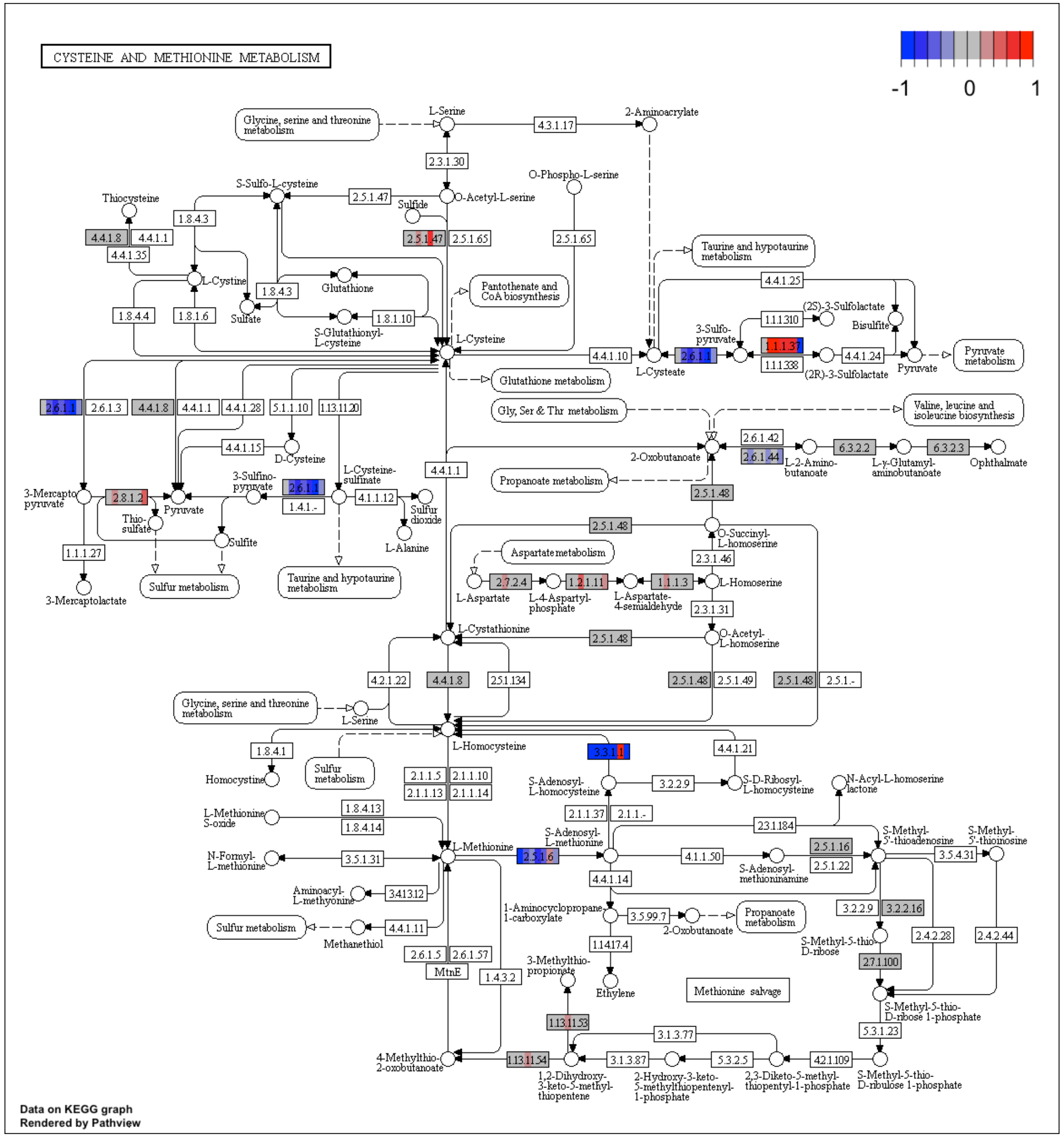

CYSTEINE AND METHIONINE METABOLISM
-1
0
1
L-Serine
2-Aminoacrylate
O-Acetyl-L-serine
O-Phospho-L-serine
S-Sulfo-L-cysteine
Thiocysteine
L-Cystine
Glutathione
Sulfate
S-Glutathionyl-L-cysteine
L-Cysteine
L-Cysteate
3-Sulfo-pyruvate
Pyruvate
Bisulfite
D-Cysteine
3-Mercaptopyruvate
3-Mercaptolactate
Thiosulfate
Sulfite
L-Alanine
2-Oxobutanoate
L-Cystathionine
L-Homocysteine
L-Homoserine
L-Aspartate
L-Methionine
S-Adenosyl-L-methionine
S-Adenosyl-L-homocysteine
Ethylene
Methionine salvage
Data on KEGG graph
Rendered by Pathview

U)

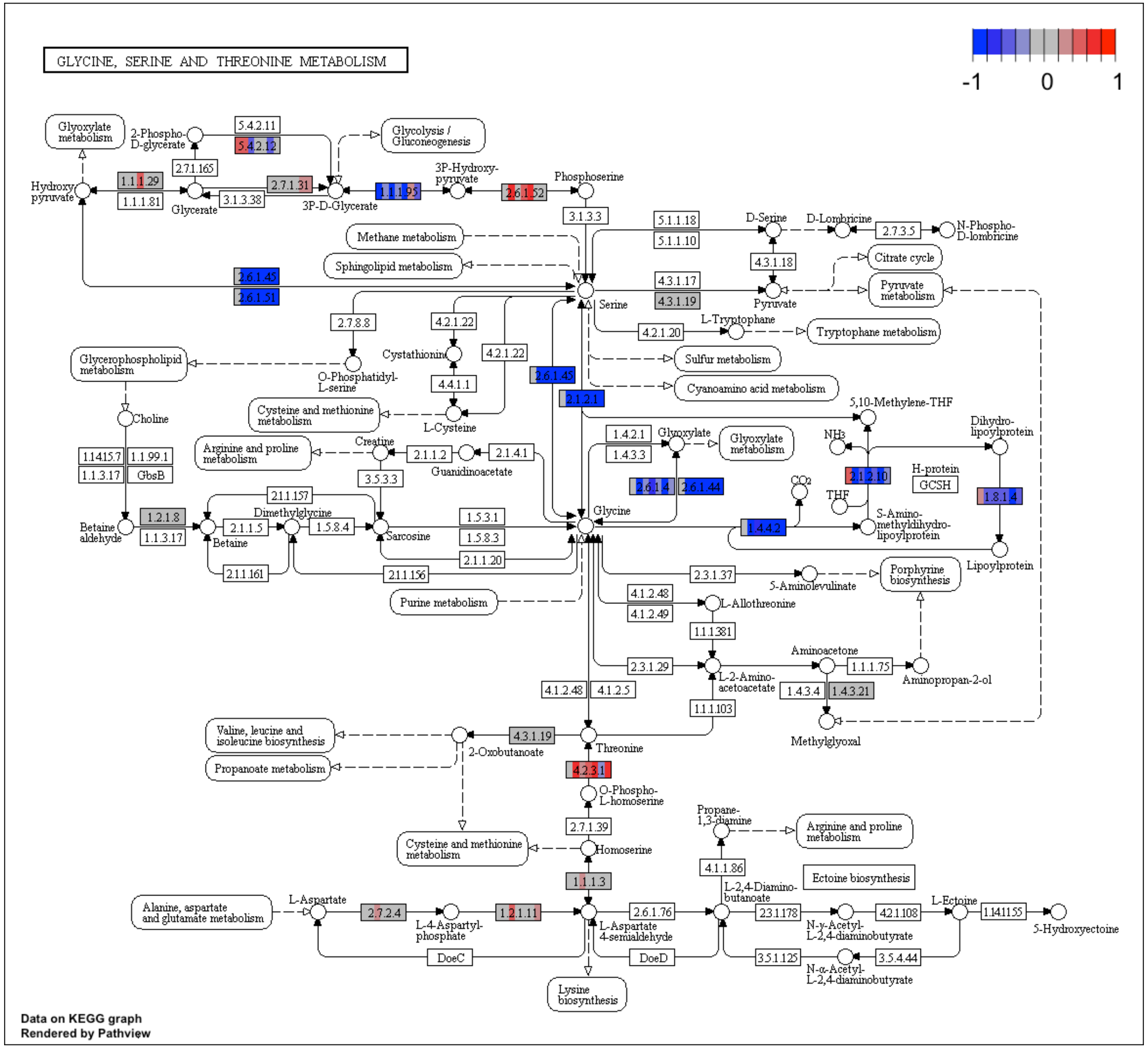
GLYCINE, SERINE AND THREONINE METABOLISM
-1
0
1
Glyoxylate metabolism
2-Phospho-D-glycerate
Glycolysis / Gluconeogenesis
3P-Hydroxy-pyruvate
Phosphoserine
Hydroxy-pyruvate
Glycerate
3P-D-Glycerate
Serine
D-Serine
D-Lombricine
N-Phospho-D-lombricine
Citrate cycle
Pyruvate metabolism
Pyruvate
Methane metabolism
Sphingolipid metabolism
L-Tryptophane
Tryptophane metabolism
Sulfur metabolism
Cyanoamino acid metabolism
Glycerophospholipid metabolism
Cystathionine
O-Phosphatidyl-L-serine
Cysteine and methionine metabolism
L-Cysteine
5,10-Methylene-THF
Choline
Arginine and proline metabolism
Creatine
Guanidinoacetate
Glyoxylate
Dihydro-lipoylprotein
H-protein
GCSH
CO2
THF
NH3
Betaine aldehyde
Dimethylglycine
Betaine
Sarcosine
Glycine
S-Amino-methyldihydro-lipoylprotein
Lipoylprotein
Porphyrine biosynthesis
5-Aminolevulinate
L-Allothreonine
Purine metabolism
Aminoacetone
Aminopropan-2-ol
L-2-Amino-acetoacetate
Methylglyoxal
Valine, leucine and isoleucine biosynthesis
Propanoate metabolism
2-Oxobutanoate
Threonine
O-Phospho-L-homoserine
Propane-1,3-diamine
Homoserine
Ectoine biosynthesis
L-Aspartate
L-4-Aspartyl-phosphate
L-Aspartate 4-semialdehyde
L-2,4-Diamino-butanoate
N-γ-Acetyl-L-2,4-diaminobutyrate
N-α-Acetyl-L-2,4-diaminobutyrate
L-Ectoine
5-Hydroxyectoine
Alanine, aspartate and glutamate metabolism
Lysine biosynthesis
5.4.2.11
5.4.2.12
2.7.1.165
1.1.1.29
1.1.1.81
3.1.3.38
2.7.1.31
1.1.1.95
2.6.1.52
3.1.3.3
5.1.1.18
5.1.1.10
2.7.3.5
4.3.1.18
4.3.1.17
4.3.1.19
2.6.1.45
2.6.1.51
2.7.8.8
4.2.1.22
4.2.1.20
4.4.1.1
2.1.2.1
1.4.2.1
1.4.3.3
2.1.1.2
2.1.4.1
3.5.3.3
2.6.1.4
2.6.1.44
2.1.2.10
1.8.1.4
1.4.4.2
1.14.15.7
1.1.99.1
1.1.3.17
GbsB
1.2.1.8
2.1.1.157
2.1.1.5
1.5.8.4
1.5.3.1
1.5.8.3
2.1.1.20
2.1.1.161
2.1.1.156
2.3.1.37
4.1.2.48
4.1.2.49
1.1.1.381
2.3.1.29
1.1.1.75
1.4.3.4
1.4.3.21
4.1.2.5
1.1.1.103
4.2.3.1
2.7.1.39
4.1.1.86
1.1.1.3
2.7.2.4
1.2.1.11
2.6.1.76
2.3.1.178
4.2.1.108
1.14.11.55
DoeC
DoeD
3.5.1.125
3.5.4.44
Data on KEGG graph
Rendered by Pathview

V)

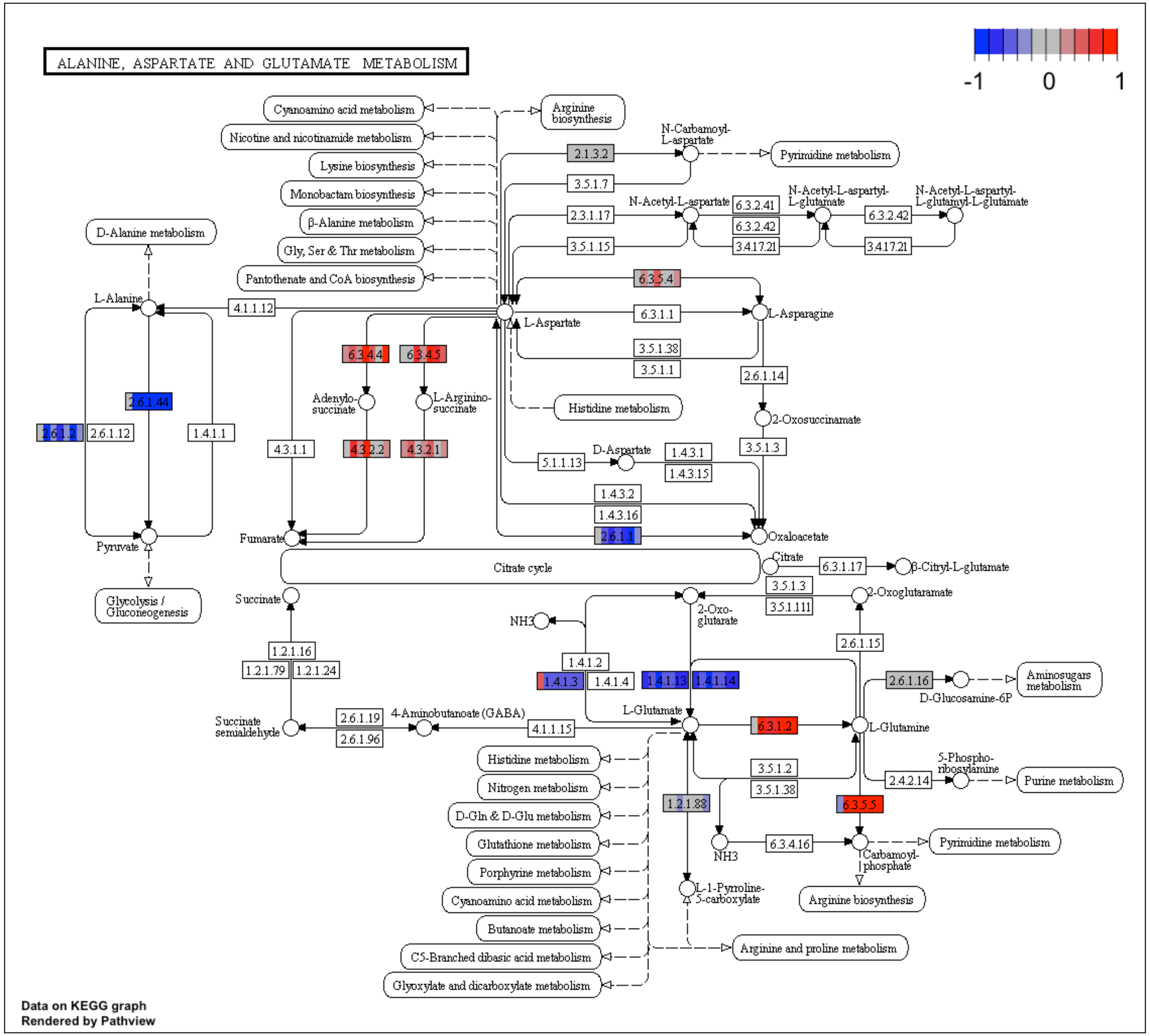
ALANINE, ASPARTATE AND GLUTAMATE METABOLISM
-1
0
1
Cyanoamino acid metabolism
Nicotine and nicotinamide metabolism
Lysine biosynthesis
Monobactam biosynthesis
β-Alanine metabolism
Gly, Ser & Thr metabolism
Pantothenate and CoA biosynthesis
Arginine biosynthesis
N-Carbamoyl-L-aspartate
Pyrimidine metabolism
2.1.3.2
3.5.1.7
2.3.1.17
3.5.1.15
N-Acetyl-L-aspartate
6.3.2.41
6.3.2.42
3.4.17.21
N-Acetyl-L-aspartyl-L-glutamate
N-Acetyl-L-aspartyl-L-glutamyl-L-glutamate
D-Alanine metabolism
L-Alanine
4.1.1.12
6.3.5.4
L-Aspartate
6.3.1.1
L-Asparagine
3.5.1.38
3.5.1.1
2.6.1.14
6.3.4.4
6.3.4.5
2.6.1.44
2.6.1.2
2.6.1.12
1.4.1.1
Adenylo-succinate
L-Arginino-succinate
Histidine metabolism
2-Oxosuccinamate
4.3.1.1
4.3.2.2
4.3.2.1
5.1.1.13
D-Aspartate
1.4.3.1
1.4.3.15
3.5.1.3
1.4.3.2
1.4.3.16
2.6.1.1
Pyruvate
Fumarate
Oxaloacetate
Glycolysis / Gluconeogenesis
Citrate cycle
Citrate
6.3.1.17
β-Citryl-L-glutamate
Succinate
3.5.1.3
3.5.1.111
2-Oxoglutaramate
NH3
2-Oxo-glutarate
1.2.1.16
1.2.1.79
1.2.1.24
1.4.1.2
1.4.1.3
1.4.1.4
1.4.1.13
1.4.1.14
2.6.1.15
2.6.1.16
D-Glucosamine-6P
Aminosugars metabolism
Succinate semialdehyde
2.6.1.19
2.6.1.96
4-Aminobutanoate (GABA)
4.1.1.15
L-Glutamate
6.3.1.2
L-Glutamine
Histidine metabolism
Nitrogen metabolism
D-Gln & D-Glu metabolism
Glutathione metabolism
Porphyrine metabolism
Cyanoamino acid metabolism
Butanoate metabolism
C5-Branched dibasic acid metabolism
Glyoxylate and dicarboxylate metabolism
3.5.1.2
3.5.1.38
5-Phospho-ribosylamine
2.4.2.14
Purine metabolism
1.2.1.88
6.3.5.5
NH3
6.3.4.16
Carbamoyl-phosphate
Pyrimidine metabolism
L-1-Pyrroline-5-carboxylate
Arginine biosynthesis
Arginine and proline metabolism
Data on KEGG graph
Rendered by Pathview
